# Deciphering the Physical Binding Mechanism of Enzyme–Photosensitizer Facilitates Catalysis-Augmented Photodynamic Therapy

**DOI:** 10.34133/research.0732

**Published:** 2025-06-03

**Authors:** Bingqing Jia, Yang Liu, Xudong Geng, Yuezheng Li, Chengmei Zhang, Yuanyuan Qu, Xiangdong Liu, Mingwen Zhao, Yanmei Yang, Weifeng Li, Yong-Qiang Li

**Affiliations:** ^1^ Institute of Advanced Interdisciplinary Science, School of Physics, Shandong University, Jinan 250100, China.; ^2^ Laboratory Animal Center of Shandong University, Jinan 250012, China.; ^3^ College of Chemistry, Chemical Engineering and Materials Science, Collaborative Innovation Centre of Functionalized Probes for Chemical Imaging in Universities of Shandong, Key Laboratory of Molecular and Nano Probes, Ministry of Education, Shandong Normal University, Jinan 250014, China.

## Abstract

Enzyme–photosensitizer (PS) conjugates hold great promise for clinical treatment of cancer and infectious diseases via catalysis-augmented photodynamic therapy (PDT). Compared to covalent coupling, physical binding utilizing noncovalent interactions provides a simple and nondestructive strategy to combine PS with enzymes. However, the mechanism of enzyme–PS physical combination remains largely unknown, and physically bonded enzyme–PS conjugates are rarely reported. Here, we systematically investigate the interacting behaviors of representative enzymes with one of the most popular PS of chlorin e6 (Ce6) and elucidate their binding dynamics and crucial determinants. Our results reveal that the positively charged and hydrophobic residues on the surface of enzymes are crucial determinants of Ce6 binding. In addition, we demonstrate that the positively charged surface area of enzymes can be employed as a reliable criterion for assessing and predicting the enzyme–Ce6 binding affinity. Guided by this criterion, we further construct catalase–Ce6 nanoconjugates (CAT–Ce6 NCs) with superior stability and robust photodynamic antimicrobial capability via physical binding. In a showcase treatment of methicillin-resistant *Staphylococcus aureus* (MRSA)-infected mouse model of subcutaneous abscess, CAT–Ce6 NCs enable hypoxia pathological microenvironment remodeling and bacteria elimination, realizing effective catalysis-augmented PDT. This study deciphers the physical binding mechanism of enzyme–PS and establishes a theoretical framework to facilitate the design and construction of outstanding enzyme–PS NCs for catalysis-augmented PDT.

## Introduction

Photodynamic therapy (PDT) is an important medical technology for targeted therapy. It combines photosensitizers (PSs) and light irradiation to generate reactive oxygen species (ROS) at the local environment of diseased cells or tissue [1,2]. Due to its unique advantages, including noninvasive, safety, and high spatiotemporal resolution, PDT has emerged as one of the most promising therapeutic strategies and attracted enormous research attentions, especially for the treatment of cancer and infectious diseases [3–5]. Despite extensive experimental research, translating PDT into clinical practice remains challenging [6,7]. Firstly, traditional PS, such as porphyrins and phthalocyanines, usually has poor solubility and photostability, resulting in very low bioavailability [8,9]. Additionally, the delivery/release of PS and the photodynamic process are highly influenced by the complicated pathological microenvironment, thereby impacting the efficacy of PDT. In particular, the severely hypoxic microenvironment, known as the “Achilles’ heel” of PDT, substantially represses the generation of ROS, which ultimately results in the failure of treatment [10,11]. To address these issues, development of innovative PDT strategies is highly desired for clinical and medical applications.

Enzyme–PS conjugates hold great promise in overcoming the limitations of traditional PDT [12]. Firstly, enzymes can improve the solubility, stability, and bioavailability of hydrophobic PS by acting biocompatible carriers [13,14]. Meanwhile, as key participants in metabolism, enzymes can refine and reconstruct the refractory pathological microenvironment via in situ biocatalysis, realizing catalysis-augmented PDT and substantially boosting the efficacy of treatment [15]. Nowadays, enzyme–PS conjugates are usually prepared by covalent coupling, which relies on complicated chemical synthesis and purification process where organic solvent and toxic reagents used would impair the activity of enzymes and frequently result in immunogenicity and inflammation [16,17]. In contrast, physical binding through noncovalent interactions provides an alternative and promising approach to combine PS with enzymes, which not only substantially simplifies and streamlines the operational process but also does not adversely impact enzyme activity [18–20]. However, physical binding is usually more complicated (involving Coulomb interaction, van der Waals force, dehydration effect, and the entropy effect) and weaker in strength than chemically covalent bonds. The present understandings of the principle of enzyme–PS physical binding remain largely unknown. A comprehensive study of enzyme–PS binding mechanism can greatly guide the design of enzyme–PS conjugates and pave the avenue of catalysis-augmented PDT in clinical and medical applications.

In this work, we systematically investigate the physical interactions of representative enzymes with a widely used PS, chlorin e6 (Ce6), by combined theoretical simulation and experimental affinity measurement. The simulation dynamics indicates that the binding of Ce6 to enzymes is firstly driven by electrostatic force to form loose contacts and stabilized by hydrophobic interaction and hydrogen bond. In addition, binding site analysis reveals that the number of positively charged and hydrophobic residues on the surface of enzymes are crucial determinants of Ce6 binding. Moreover, we demonstrate that the positively charged region area on the surface of enzyme can be employed as a reliable criterion for predicting the enzyme–Ce6 binding affinity. Guided by this criterion, we further construct catalase–Ce6 nanoconjugates (CAT–Ce6 NCs), which show high binding affinity, superior stability, and robust photodynamic antimicrobial capability in hypoxic condition. In methicillin-resistant *Staphylococcus aureus* (MRSA)-infected mouse model of subcutaneous abscess, the CAT–Ce6 NCs enable hypoxia pathological microenvironment remodeling and bacteria elimination, realizing efficient catalysis-augmented PDT. This study establishes a theoretical framework to elucidate the physical binding of enzyme–PS and provides a straightforward strategy to prepare enzyme–PS NCs and facilitate catalysis-augmented PDT.

## Results

### Theoretical simulation of physical interaction between enzymes and Ce6

**Fig. 1.**
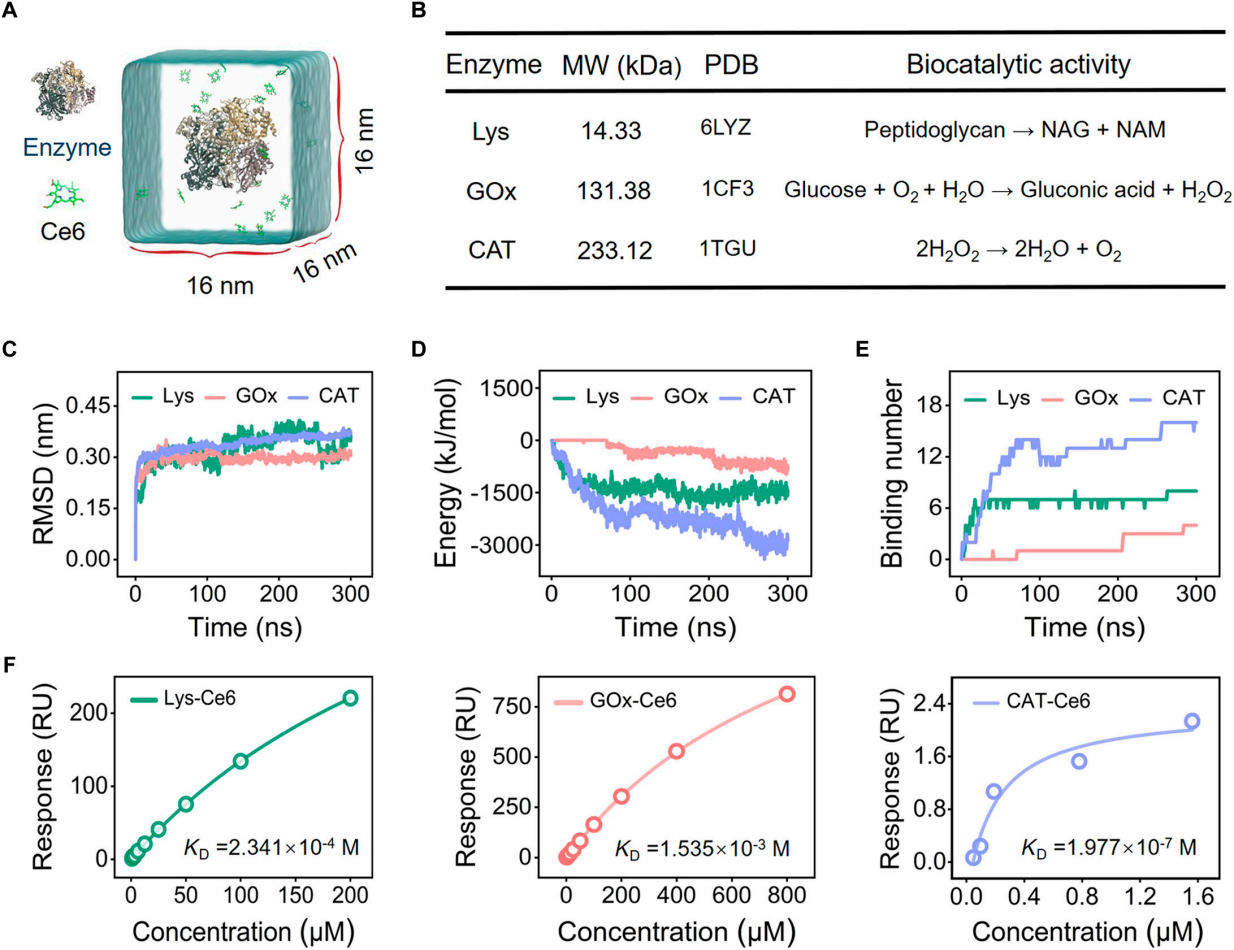
Theoretical simulation of physical interaction between enzymes and Ce6. (A) Schematic illustration of the theoretical model of MD simulation for enzyme–Ce6 physical interaction. (B) Selected representative enzymes and the information of their molecular weight (MW), Protein Data Bank (PDB) number, and biocatalytic activity (NAG, N-acetylglucosamine; NAM, N-acetylmuramic acid). (C) Time evolution of the RMSD of heavy atoms of enzymes during simulation. (D) Time evolution of interaction energy between enzymes and Ce6 during simulation. (E) Time evolution of the contact number of Ce6 to enzymes during simulation. (F) *K*_D_ of enzyme–Ce6 to indicate the experimental affinity of Ce6 to enzymes.

Molecular dynamics (MD) simulations are important tools for understanding the structure of biological macromolecules and the physical basis [21,22]. Thus, in the present work, MD simulations were firstly employed to theoretically explore the physical interaction between enzymes and Ce6 before conducting experiments. Figure 1A shows the theoretical model of MD simulation in which one enzyme and 20 Ce6 molecules were solvated in an aqueous environment as the initial configurations to ensure a comparable concentration of Ce6 among all the simulated systems. Here, 3 typical enzymes including lysozyme (Lys), glucose (GOx), and CAT with different molecular weights and biocatalytic activities were chosen as representatives with the aim of deriving general conclusions (Fig. 1B). All MD simulations were conducted using the GROMACS2019 package, and the GROMOS united-atom force field was applied to describe the interactions in the simulation systems. Given that all enzyme–Ce6 complex simulation systems in preliminary experiments reached stabilization at approximately 200 ns, we opted for a simulation duration of 300 ns to ensure the accuracy of MD results. Moreover, for each enzyme–Ce6 complex, 3 independent simulations were conducted to ensure efficient sampling.

We first checked the structural stability of enzymes during simulation. Figure 1C showed that the root mean square deviation (RMSD) of enzymes’ heavy atoms with respect to their initial state (the nuclear magnetic resonance structure) reached a plateau quickly after the beginning of the simulation. In addition, the secondary structure of enzymes was also found to remain highly intact during simulation, showing no substantial damage or loss (Figs. S1 to S3). These results suggested that the enzymes maintained stable structures throughout the simulation. Subsequently, the time evolution of the interaction energy between enzymes and Ce6 molecules was analyzed. As depicted in Fig. 1D and Fig. S4, at the end of simulation, the interaction energy reached −2,867.37, −1,456.13, and −748.88 kJ/mol for Ce6 binding with CAT, Lys, and GOx, respectively, indicating that Ce6 is inclined to bind to CAT more easily than do Lys and GOx. Furthermore, the contact number of Ce6 to the 3 enzymes at the end of simulation reached 16, 8, and 6 for CAT, Lys, and GOx, respectively, showing a consistent trend with the interaction energy results (Fig. 1E and Fig. S4). From the above simulation results, we could conclude that the binding affinity of Ce6 to enzymes follows the order of CAT > Lys > GOx.

To validate this theoretical prediction, surface plasmon resonance (SPR) analysis was performed to evaluate the affinity between enzymes and Ce6 [23]. As shown in Fig. 1F and Fig. S5, the equilibrium dissociation constants (*K*_D_) of CAT–Ce6, Lys–Ce6, and GOx–Ce6 were determined to be 1.977 × 10^−7^, 2.341 × 10^−4^, and 1.535 × 10^−3^ M, respectively, indicating that the experimental affinity of Ce6 to enzymes follows the same order with the theoretical binding energy. These findings demonstrated that MD simulation could accurately represent the actual information of physical interactions between enzymes and Ce6, laying a solid foundation for subsequent physical binding mechanism investigation.

### The binding dynamics and binding site analysis of enzyme–Ce6

**Fig. 2.**
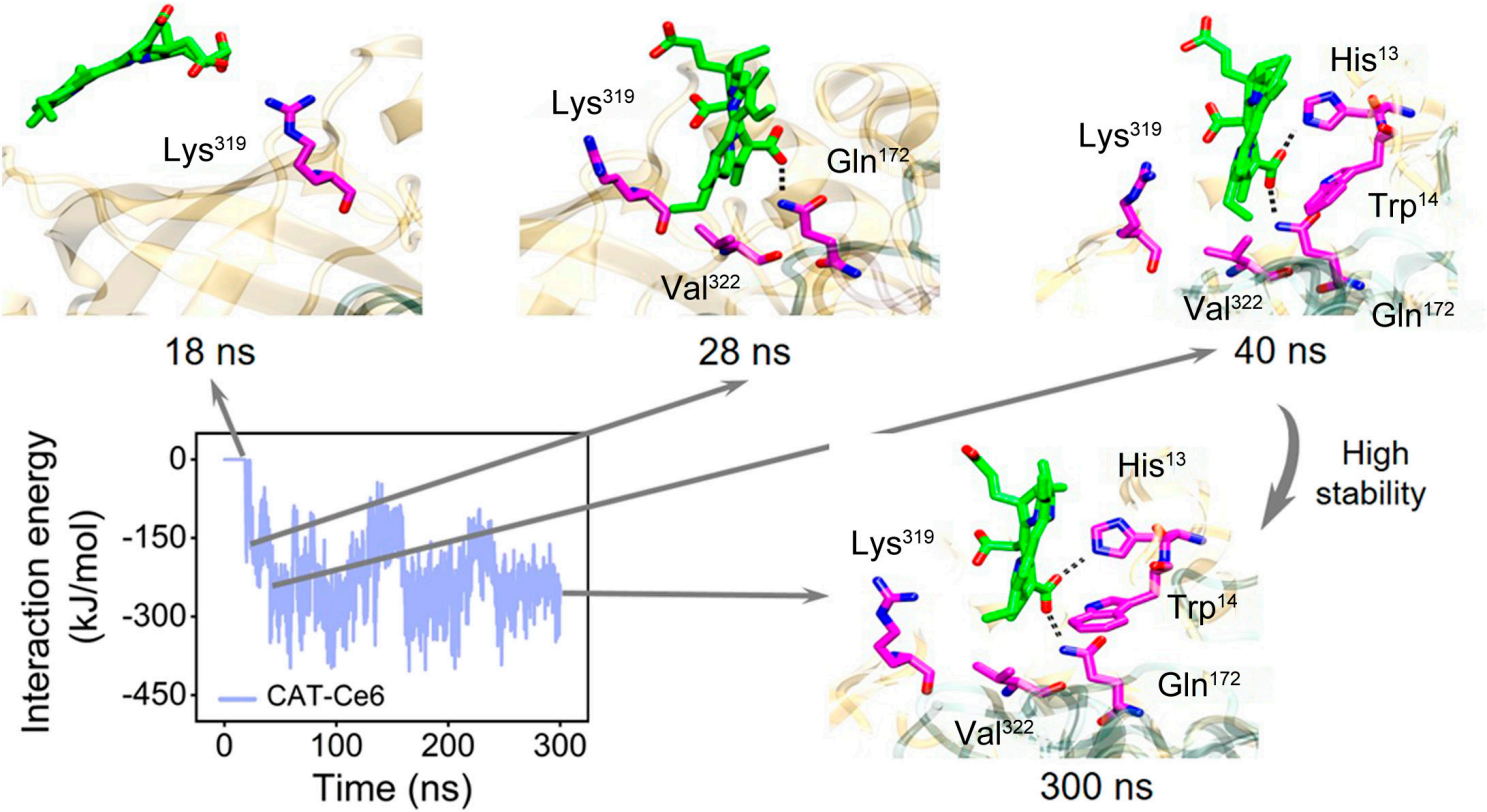
The binding dynamics of CAT–Ce6. The interaction energy during 300-ns simulation of CAT–Ce6 binding is calculated, and the critical residues during the binding process are depicted. Dashed lines represent hydrogen bonds formed between CAT and Ce6.

Given that Ce6 showed the highest binding affinity to CAT, we selected one trajectory of CAT–Ce6 as the representative to address the dynamic process of enzyme–Ce6 binding and the underlying working mechanism. The binding process was firstly investigated through focusing on one Ce6 molecule binding with CAT. Figure 2 shows the time evolution of interaction energy with 4 representational conformations during the whole binding process. More specifically, the residues of CAT that interact directly with Ce6 were highlighted. At the simulation beginning, the Ce6 freely diffused in the aqueous solvent about 18 ns, after which the Ce6 gradually approached the CAT surface due to the electrostatic attraction from Lys^319^, resulting in a clear decrease in the interaction energy. At 28 ns, Ce6 further engaged in a hydrophobic interaction with Val^322^ and formed one hydrogen bond with Gln^172^, forming a meta-stable binding complex. During the subsequent simulation, the Ce6 adjusted its orientation to establish more contacts with Trp^14^ and the second hydrogen bond with His^13^ at 40 ns. During the rest of the simulation, the Ce6 molecule remained such binding site with CAT, revealing a very stable binding pattern. To verify the stability of the local conformation following the binding of CAT with Ce6, we calculated the mean root mean square fluctuation (RMSF) for the residues in the binding pocket during the final 100 ns of simulation. As shown in Fig. S6, the RMSF values for all residues within the binding pocket were minimal, implying that the conformation of the CAT–Ce6 complex persisted in a stable state post-binding.

**Fig. 3.**
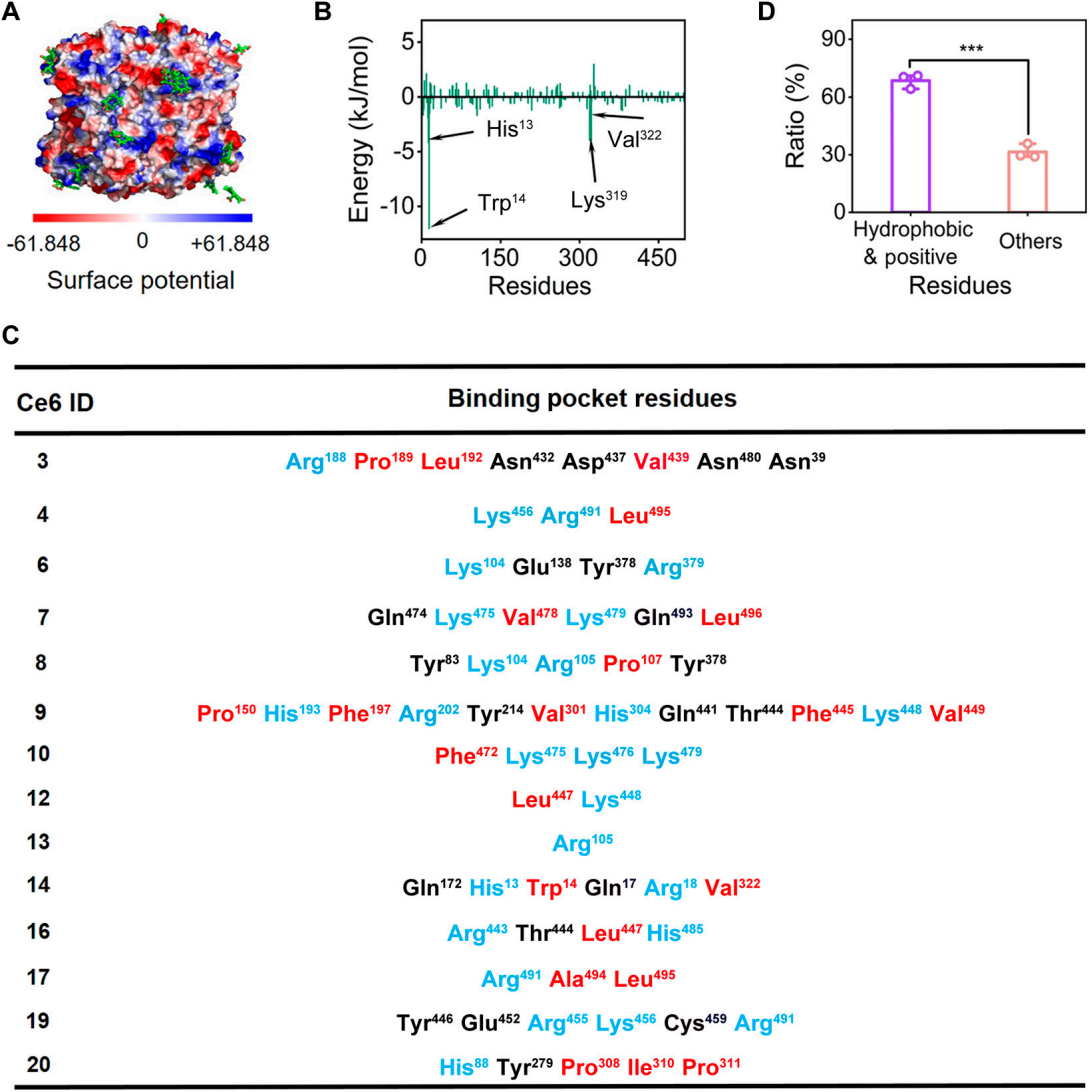
Binding site analysis of CAT–Ce6. (A) Surface electrostatic potential map of CAT–Ce6. The red and blue colors represent the negatively charged region and positively charged region, respectively. (B) Contribution of residues to the binding energy of CAT–Ce6. The black arrows indicate that the residues contributed the most. (C) Composition of residues in each binding site of CAT–Ce6. Blue represents positively charged residues, and red represents hydrophobic residues. (D) Statistical analysis of the ratio of hydrophobic and positively charged residues to the total number of residues in all of the binding site of CAT–Ce6. The values of ratio represent the mean of 3 independent experiments, and the error bars indicate the standard deviation (SD) from the mean. ***P < 0.001.

From the above analysis, it is highly expected that the positively charged residues on the CAT surface play a key role in the physical binding of Ce6 with CAT. To validate this observation, we draw the surface electrostatic potential map of CAT–Ce6 and the positions of the Ce6 molecules. As depicted in Fig. 3A, all the Ce6 bound in predominantly positive-charged pockets. The same phenomenon was also observed for the binding of Ce6 with GOx and Lys (Fig. S7). The significance of positive-charged residues may lie in 2 aspects: (a) initiate the binding process through electrostatic attractions and (b) enhance the complex stability by forming hydrogen bonds with Ce6. Thus, to quantify the contribution of various residues to the binding strength of CAT–Ce6, the total binding free energy of CAT–Ce6 was calculated and deposited to each residue using the Poisson–Boltzmann surface area (MM-PBSA) method [24]. As shown in Fig. 3B, the top 4 residues contributing to the binding of CAT–Ce6 were identified to be Trp^14^ (−12.04 kJ/mol), His^13^ (−4.21 kJ/mol), Val^322^ (−4.00 kJ/mol), and Lys^319^ (−3.97 kJ/mol). These results suggested that the positively charged and hydrophobic (especially those of the aromatic species) residues on the CAT surface indeed played an essential role for the binding of CAT–Ce6. To further verify these preliminary findings, we calculated the contact map of Ce6 with CAT (Fig. S8). Here, those residues with a contacting probability larger than 70% with Ce6 were treated as contacting residues, which were summarized in Fig. 3C. It was clear that the positively charged and hydrophobic residues were dominant binding residues to Ce6. More specifically, they constituted approximately 68% to the total 14 binding sites for Ce6 (Fig. 3D). This was expected because Ce6 had a negatively charged and hydrophobic coexisting surface. The 3 carboxyl groups could form intimate interactions with CAT by forming hydrogen bonds. Meanwhile, the hydrophobic parts could further stabilize the CAT–Ce6 binding through the dehydration effect. The same trend was also observed in the binding of GOx–Ce6 and Lys–Ce6 (Figs. S9 and S10 and Tables S1 and S2). Thus, it is highly speculated that the essential role of these 2 types of residues in the Ce6–enzyme binding is universal.

To verify this speculation, we further simulated 3 more enzymes including horseradish peroxidase (HRP), alkaline phosphatase (ALP), and lactate dehydrogenase A (LDHA), which not only cover a large range of molecular weight but also have broad biomedical applications [25–27]. The contact maps of the 3 enzymes with Ce6 molecules also supported the conclusion of positively charged and hydrophobic residues on the surface of enzyme being crucial determinants for Ce6 binding (Figs. S11 to S14 and Tables S3 to S5). However, it was worth mentioning that for small molecular weight enzymes of Lys (11.33 kDa) and HRP (34.84 kDa), we observed aggregation events of 2 Ce6 molecules through the π–π interaction on the enzyme surface (Figs. S15 and S16). It is well known that the aggregation of PS could lead to the quenching and deactivation of PS, resulting in the suppression of ROS generation and the PDT treatment [28]. The Lys and HRP interacting with Ce6 may not follow a general rule of enzyme–Ce6 physical binding, because they are too small to provide enough space for Ce6 binding. Therefore, the binding dynamics of Lys–Ce6 and HRP-Ce6 were not used in the following analysis.

### Criterion for predicting the binding strength of enzyme–Ce6

**Fig. 4.**
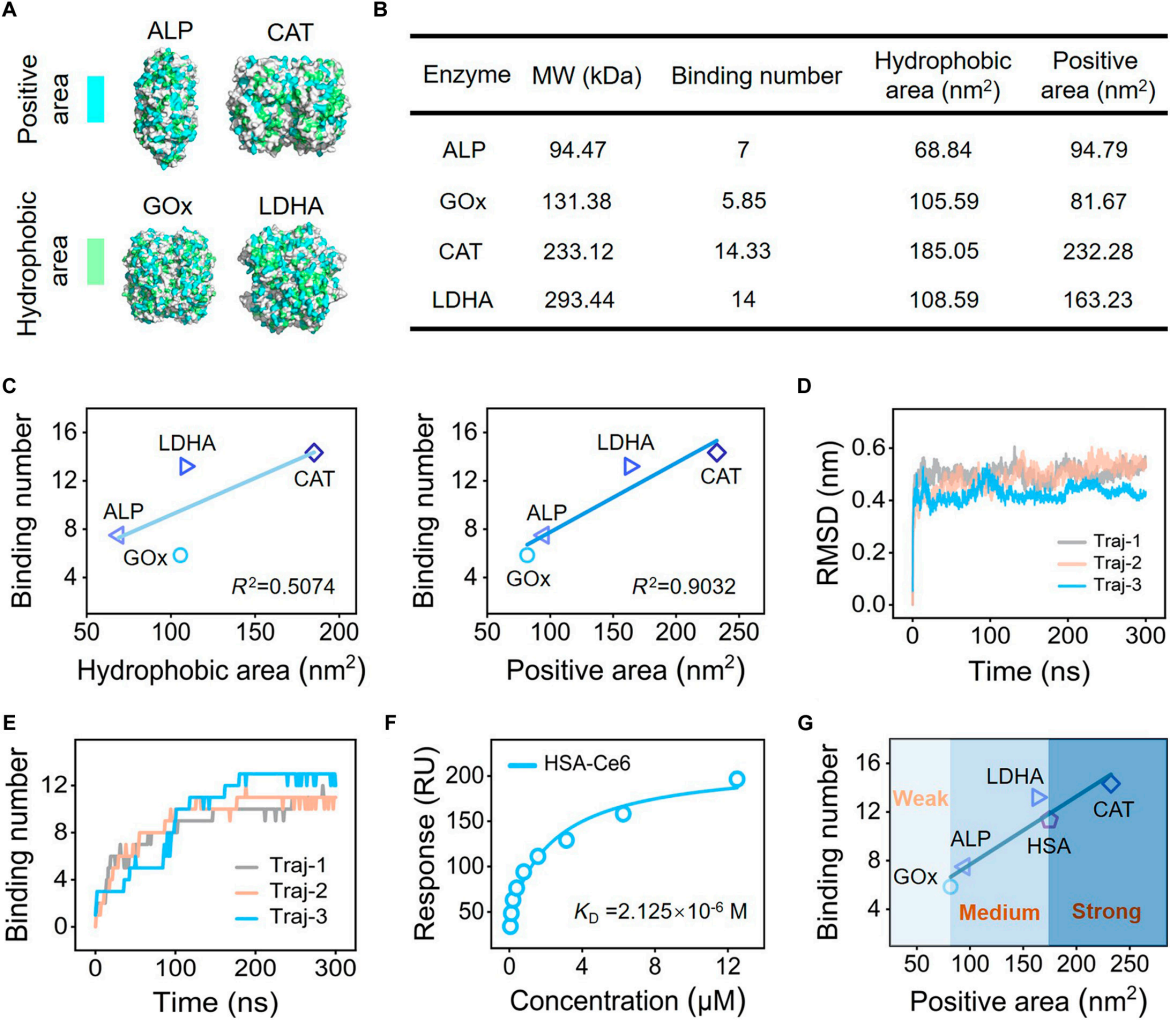
Criterion for predicting the binding strength of enzyme–Ce6. (A) Colored position of positively charged and hydrophobic residues on the surface of ALP, GOx, CAT, and LDHA. (B) Calculated surface positive and hydrophobic areas of ALP, GOx, CAT, and LDHA, and the corresponding binding number of Ce6. (C) Correlation between the total binding number of Ce6 to enzymes and the surface area of hydrophobic and positively residues. (D) Time evolution of the RMSD of heavy atoms of HSA during simulation. (E) Time evolution of the contact number of Ce6 to HSA during simulation. (F) *K*_D_ of HSA-Ce6 to indicate the experimental affinity of Ce6 to HSA. (G) Proposed criterion for predicting the binding affinity of enzyme–Ce6 based on the area of positively charged regions on the surface of enzyme.

The above findings provide an important clue for predicting the binding strength of enzyme–Ce6 by characterizing the enzyme surface, more specifically, the positively charged and hydrophobic residues’ area. Considering this, we colored the surface residues of ALP, GOx, CAT, and LDHA, according to the charge/hydrophobicity (Fig. 4A), and calculated the total area for each species (Fig. 4B and Fig. S17). The correlation between the total binding number of Ce6 to the enzymes and the surface area of positively charged and hydrophobic residues was summarized in Fig. 4C. It was clear that there was poor correlation (*R*^2^ = 0.5074) between the area of hydrophobic region of enzymes and the Ce6 binding number. Surprisingly, the Ce6 binding number (*y*) showed a strong correlation (*R*^2^ = 0.9032) with the area of positively charged region (*x*) with a fitted linear function of *y* = 0.057*x* + 2.051. Based on the results shown in Fig. 2, free Ce6 molecule initially attached to the enzyme surface through electrostatic attractions, after which hydrophobic interactions further anchored Ce6. However, compared to hydrogen bonds, the hydrophobic interactions lack specificity. Therefore, the area of positively charged residues is believed to be a better indicator for assessing Ce6 binding.

Because most enzymes are proteins, the above criterion is principally applicable to Ce6 binding with other proteins. Considering the universal existence and the high concentration of human serum albumin (HSA) in the bloodstream [29,30], it inevitably forms complex with Ce6 during intravenous administration, competing with enzymes. Thus, the HSA and Ce6 binding was chosen as a prototype validation of the criteria. We adopted the same procedure in HSA simulation with Ce6. MD simulations indicated that HSA possessed high stability during the physical interactions with Ce6, as was evident by the fast-balanced heavy atoms’ RMSD and highly intact secondary structure of HSA (Fig. 4D and Fig. S18). In addition, the interaction energy between Ce6 and HSA gradually decreased and ultimately converged during simulation, indicating that HSA could form stable bindings with Ce6 (Fig. S19). The binding number of Ce6 bound to HSA reached about 11.33 at the end of simulation (Fig. 4E). Furthermore, the *K*_D_ of HSA-Ce6 was calculated to be 2.125 × 10^−6^ M based on SPR analysis, which was consistent with the MD simulation results and confirmed the high affinity of HSA-Ce6 (Fig. S20 and Fig. 4F). More importantly, positively charged residues were uniformly found in all the binding sites of Ce6, indicating the strong dependence between the positively charged region of HSA and the binding affinity of HSA-Ce6 (Fig. S21 and Table S6).

Based on all the simulated enzyme–Ce6 data, the relationship was obtained as:

N=0.05599S+2.08294 (1)

where N is the Ce6 binding number and *S* denotes the area of positively charged region of enzymes (Fig. 4G). *R*^2^ reached 0.898, indicating high reliability of the dependence. Based on this relationship, we proposed a criterion for rapid assessing and predicting the binding affinity of enzyme–Ce6 by using the area of positively charged regions on the surface of enzyme. As shown in Fig. 4G, the binding strength of Ce6 to enzyme was divided into 3 categories—weak, medium, and strong—according to the area of positively charged region on the enzyme surface. Firstly, it is well known that the interaction between the ligand and protein is weak with nonspecificity when the *K*_D_ exceeds 10^−3^ M [31]. Meanwhile, the *K*_D_ of GOx–Ce6 in our experiments was determined to be 1.535 × 10^−3^ M. Therefore, GOx–Ce6 was treated as the boundary of weak and medium interacting regions where the corresponding area value is 81.67 nm^2^. In contrast, a *K*_D_ of less than 10^−6^ M reveals highly stable binding of host–guest complexes [32]. Considering that the HSA-Ce6 has a *K*_D_ of 2.125 × 10^−6^ M, the corresponding area of 174.45 nm^2^ was used as the boundary between medium and strong interactions. Last, if the *S* of certain enzyme exceeds 174.45 nm^2^, the binding to Ce6 could be deemed strong binding. In this scenario, the enzyme–Ce6 complex could form a structured entity that enhances the bioavailability of the PS and facilitates adaptation to harsh pathological microenvironments, achieving ultimate catalysis-augmented PDT.

### Construction and characterization of CAT–Ce6 NCs

**Fig. 5.**
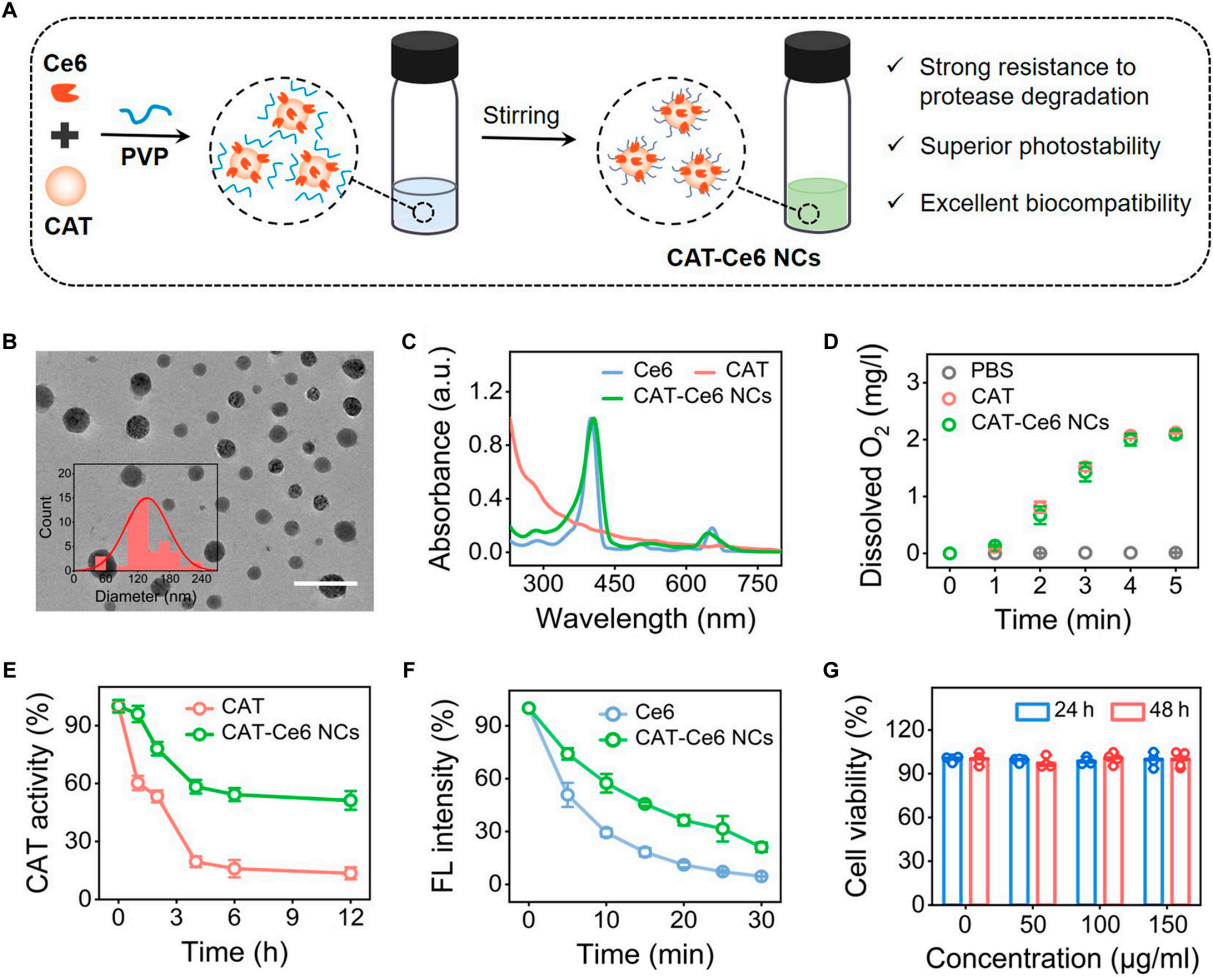
Preparation and characterization of CAT–Ce6 NCs. (A) Schematic illustration of CAT–Ce6 NC preparation. (B) Representative TEM image and corresponding size distribution (inset) of CAT–Ce6 NCs. Scale bar, 500 nm. (C) UV-Vis absorption spectra of Ce6, CAT, and CAT–Ce6 NCs. (D) Time evolution of dissolved O_2_ concentration of PBS, CAT, and CAT–Ce6 NC solution during 5-min incubation with H_2_O_2_ (1 × 10^−4^ M). (E) Time evolution of CAT activity of free CAT and CAT–Ce6 NCs during 12-h incubation with proteinase K (20 μg/ml). (F) Time evolution of fluorescence intensity of Ce6 and CAT–Ce6 NCs during 30-min laser irradiation (660 nm, 0.8 W/cm^2^). (G) Viability of HUVECs after incubation with CAT–Ce6 NCs at various concentrations for 24 and 48 h. In (D) to (G), data are presented as the mean ± SD (*n* = 3 independent experiments).

CAT is a core antioxidant enzyme in most organisms that catalyzes the decomposition of hydrogen peroxide (H_2_O_2_) to generate O_2_, and has been considered as a robust candidate to combat the hypoxia limit of PDT [33,34]. According to the criterion of the positively charged region area we proposed, CAT has strong binding with Ce6 and it is wonderful to construct CAT–Ce6 conjugates via simple physical binding and facilitate catalysis-augmented PDT. However, previous works reported that enzyme–PS complexes were easily susceptible to protease degradation and photobleaching in various application scenarios, limiting their application prospect [35,36]. Nanocrystallization has been shown to be an effective strategy to improve the stability of traditional protein drug and PSs, and shield them from the influence of proteases and photoirradiation [37–39]. Therefore, we constructed CAT–Ce6 NCs with superior stability to resist protease hydrolysis and photobleaching by exploiting the strong affinity between CAT and Ce6 and the nanostructure-directing effect of polyvinylpyrrolidone (PVP) (Fig. 5A) [40]. Transmission electron microscopy (TEM) and scanning electron microscopy (SEM) images showed that CAT–Ce6 NCs had regular and well-defined spherical nanostructures with an average size of 137.34 ± 40.34 nm (Fig. 5B and Fig. S22). The ultraviolet–visible (UV-Vis) absorption spectrum showed the characteristic absorption peaks of Ce6 at round 400 and 660 nm in the CAT–Ce6 NCs, and the fluorescence emission wavelengths of CAT–Ce6 NCs and free Ce6 were similar under 620 nm excitation, confirming the presence of Ce6 (Fig. 5C and Fig. S23). In addition, the scanning transmission electron microscopy (STEM) and energy-dispersive spectroscopy (EDS) images of CAT–Ce6 NCs revealed that the characteristic sulfur element of CAT was clearly observed, indicating the presence of CAT (Fig. S24). Moreover, identical protein bands for both free CAT and CAT–Ce6 NCs were identified from the sodium dodecyl sulfate–polyacrylamide gel electrophoresis (SDS-PAGE) analysis, confirming the successful incorporation of CAT in the synthesized CAT–Ce6 NCs (Fig. S25). The amount of Ce6 and CAT in CAT–Ce6 NCs (1 mg of CAT–Ce6 NCs contained 0.106 mg of Ce6 and 0.894 mg of CAT) was quantitatively determined based on the standard absorption curve of Ce6 (Fig. S26) and bicinchoninic acid (BCA) assay (Fig. S27), respectively. Subsequently, CAT–Ce6 NCs were introduced into the H_2_O_2_ solution and their CAT catalytic activity was evaluated through the measurement of dissolved O_2_. As shown in Fig. 5D, CAT–Ce6 NCs generated a substantial amount of dissolved O_2_, while the phosphate-buffered saline (PBS) group exhibited no such production, indicating the notable CAT activity of CAT–Ce6 NCs. Furthermore, the production of dissolved O_2_ by both CAT and CAT–Ce6 NCs was essentially identical, indicating that the catalytic activity of CAT was not impaired during the preparation of CAT–Ce6 NCs due to the nondestructive nature of physical binding.

To assess the performance of CAT–Ce6 NCs to resist protease degradation, their CAT activity was evaluated in the presence of proteinase incubation. As shown in Fig. 5E, CAT–Ce6 NCs retained more than 60% of their CAT activity after 4-h incubation of proteinase K, while the activity of free CAT rapidly decreased to 20%. This phenomenon suggested that CAT–Ce6 NCs had substantially enhanced resistance to proteinase degradation compared to free CAT due to the protection of nanostructures. In addition, we investigate the photostability of CAT–Ce6 NCs under laser irradiation to assess their capability to resist photobleaching. As shown in Fig. 5F, the fluorescence intensity of CAT–Ce6 NCs substantially exceeded that of free Ce6 during the continuous 30-min laser irradiation, indicating that the formation of nanosized CAT–Ce6 enhanced the photostability of Ce6, aligning with our initial expectations. Additionally, CAT–Ce6 NCs exhibited the optical catalytic activity at pH 7 and 45 °C, and possessed enhanced enzymatic activity compared to free CAT across a range of pH and temperature conditions, showing broad application prospects in biological systems (Fig. S28).

By considering the negligible cytotoxicity of CAT and Ce6, CAT–Ce6 NCs were anticipated to demonstrate excellent biocompatibility. To confirm this, the viability of human umbilical vein endothelial cells (HUVECs) and NIH/3T3 cells was firstly investigated after incubation with CAT–Ce6 NCs. As shown in Fig. 5G and Fig. S29, HUVECs and NIH/3T3 cells maintained a survival rate exceeding 98% after incubation with different concentrations of CAT–Ce6 NCs for 48 h, indicating the excellent biosafety of CAT–Ce6 NCs in vitro. In addition, blood routine of CAT–Ce6 NC-administrated mice was within normal ranges, and no abnormalities in major organs were identified, indicating negligible systemic toxicity in vivo (Figs. S30 and S31). Strong resistance to protease degradation, superior photostability, and undetectable biotoxicity energize CAT–Ce6 NCs as potent drugs for catalysis-augmented PDT.

### Photodynamic antimicrobial capability of CAT–Ce6 NCs in hypoxic condition

**Fig. 6.**
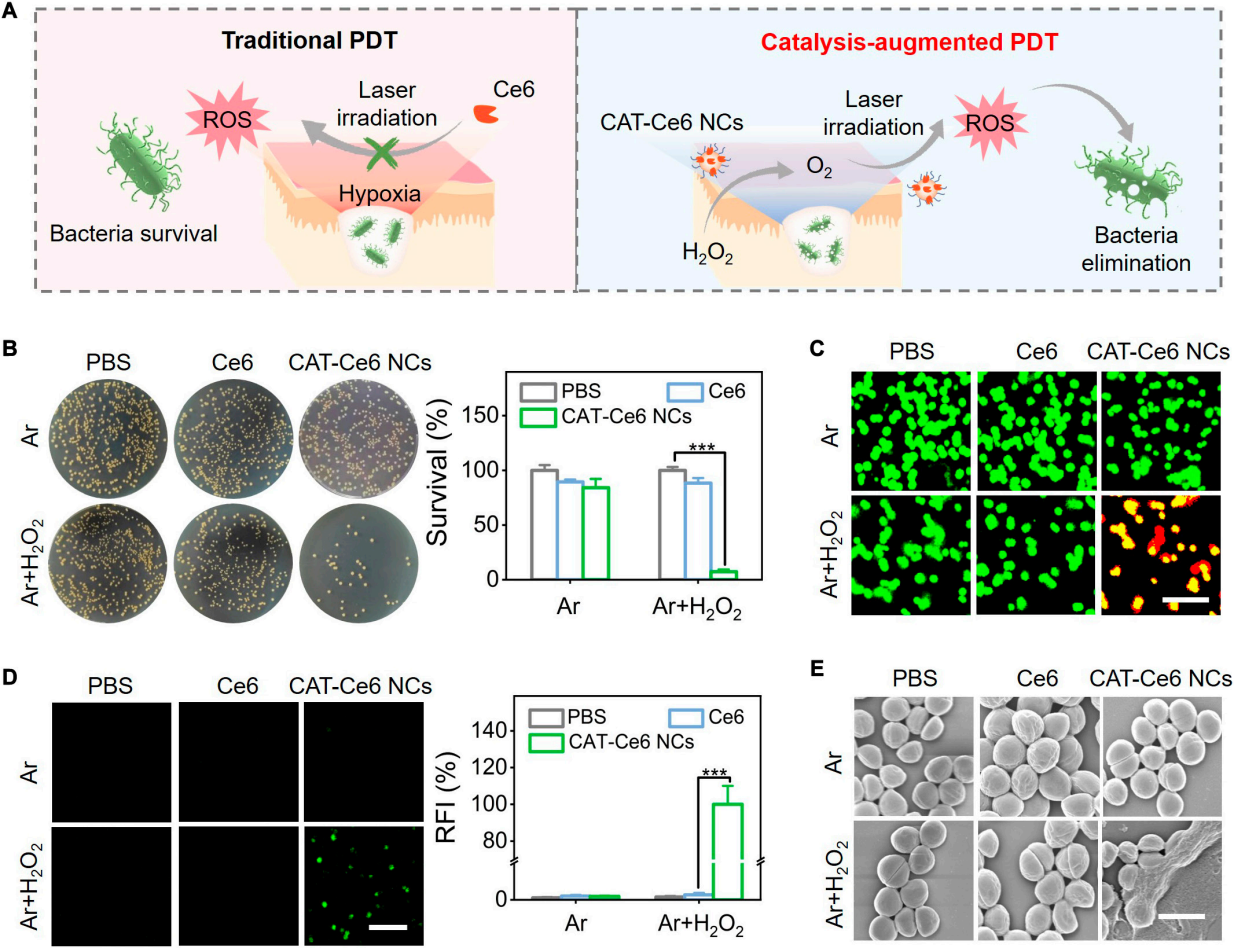
Photodynamic antimicrobial capability of CAT–Ce6 NCs in hypoxic condition. (A) Schematic illustration of traditional PDT and CAT–Ce6 NC-based catalytic-augmented PDT for subcutaneous bacteria elimination. (B) Photographs of bacterial colonies and the corresponding quantitative survival rate in different treatment groups. (C) Confocal images of live/dead bacterial staining assay in different treatment groups in which live and dead bacteria emitted green and red fluorescence, respectively. Scale bar, 10 μm. (D) Fluorescence (green) images of intracellular ROS level and the corresponding semiquantitative statistics of relative fluorescence intensity (RFI) statistics in different treatment groups. Scale bar, 10 μm. (E) SEM-based morphology observation of bacteria in different treatment groups. Scale bar, 1 μm. In the above experiments, 2 hypoxic conditions (Ar and Ar + H_2_O_2_) were constructed, and laser irradiation (660 nm, 0.8 W/cm^2^, 10 min) was employed. In (B) and (D), data are presented as the mean ± SD (*n* = 3 independent experiments). ^***^P < 0.001.

In recent decades, bacterial infections have become a substantial threat to public health, and the emergence of drug-resistant bacteria compels the search for innovative antimicrobial modalities [41,42]. PDT presents a promising therapeutic strategy for bacterial infections due to their robust antimicrobial capability toward multidrug-resistant bacteria [43,44]. However, traditional PDT is limited for treating subcutaneous infections due to the severely hypoxic pathological microenvironment, which greatly hinders the generation of bactericidal ROS [45]. H_2_O_2_ is overproduced in the bacterial microenvironment by bacteria metabolism and macrophages [46]. By decomposing H_2_O_2_ to O_2_ and water via CAT catalysis, CAT–Ce6 NCs is expected to provide a catalytic-augmented PDT strategy for alleviating hypoxia and producing ROS to eliminate subcutaneous bacteria (Fig. 6A). To confirm this, photodynamic antimicrobial capability of CAT–Ce6 NCs in hypoxia condition (Ar environment) with H_2_O_2_ incubation was evaluated. Here, gram-positive MRSA and gram-negative *Escherichia coli* were selected as the model pathogenic bacteria. As shown in Fig. 6B and Fig. S32A, the limited photodynamic antibacterial efficacy of Ce6 in Ar environment without/with H_2_O_2_ incubation implied that traditional PDT was ineffective in hypoxic condition. CAT–Ce6 NCs showed similar bactericidal performance with free Ce6 in Ar environment without H_2_O_2_ incubation, but their antimicrobial rates substantially increased in Ar environment with H_2_O_2_ incubation under the optimal PDT conditions (660 nm, 0.8 W/cm^2^, 10 min), showing incontestable catalysis-augmented photodynamic antimicrobial performance (Fig. 6B and Figs. S32A and S33). The live/dead bacterial staining results further confirmed the desired photodynamic antimicrobial capability of CAT–Ce6 NCs in hypoxic condition, where almost all bacteria were eliminated (red fluorescence) in Ar environment with H_2_O_2_ incubation (Fig. 6C and Fig. S32B). Subsequently, bacterial intracellular ROS staining and SEM-based bacterial morphology study were performed to investigate the antimicrobial mechanism of CAT–Ce6 NCs. As shown in Fig. 6D and Fig. S32C, by using a 2′,7′-dichlorofluorescein diacetate (DCFH-DA) probe, bright intracellular green fluorescence was only observed in the CAT–Ce6 NC group (with H_2_O_2_ incubation) under Ar condition, demonstrating an ROS burst caused by the CAT catalysis-augmented photodynamic process. ROS burst would result in oxidative stress and severe structure damage of bacteria to kill them [47,48]. Substantial cellular deformation and surface collapse of bacteria were clearly observed via SEM images in the group of CAT–Ce6 NCs under Ar condition and H_2_O_2_ incubation, suggesting a cell wall and membrane disruption-involved bactericidal mechanism of CAT–Ce6 NCs (Fig. 6E and Fig. S32D). In conclusion, by combining the catalytic activity of CAT and photodynamic capability of Ce6, CAT–Ce6 NCs can effectively catalyzed the decomposition of H_2_O_2_ to produce O_2_, which can improve the hypoxic pathological microenvironment and further promote the production of ROS via photodynamic process under laser irradiation. The produced ROS can further oxidize and compromise the structural integrity of bacterial cell wall and membrane, ultimately achieving catalysis-augmented PDT of bacterial infection. Remarkably, CAT–Ce6 NCs were found to have outstanding antimicrobial activity comparable to super antibiotics of vancomycin (Figs. S34 and S35). This exceptional photodynamic antimicrobial capability of CAT–Ce6 NCs in hypoxic condition lays a solid foundation for subcutaneous bacterial infection treatment.

### In vivo catalysis-augmented PDT of MRSA-infected subcutaneous abscess

**Fig. 7.**
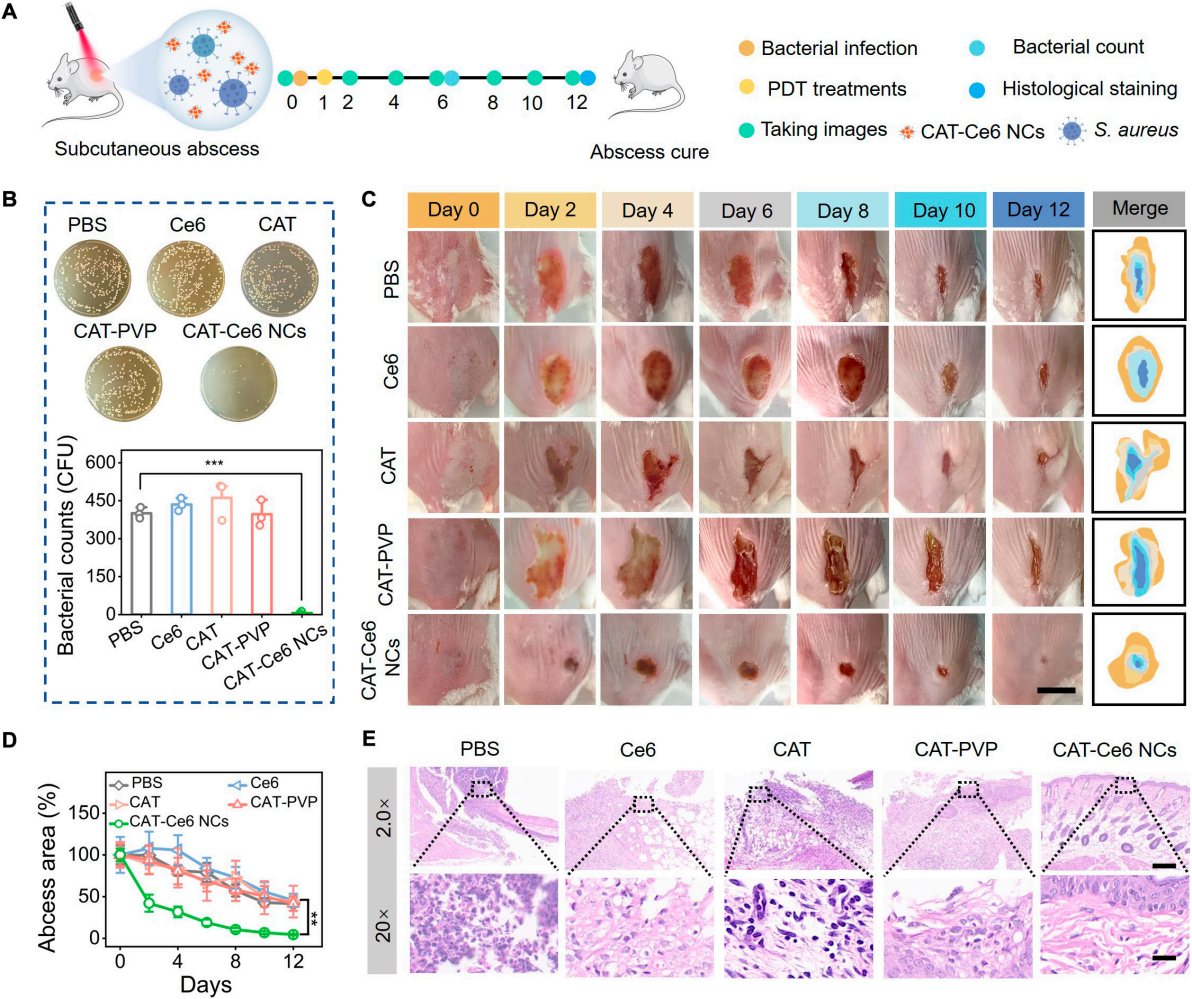
In vivo catalysis-augmented PDT of MRSA-infected subcutaneous abscess. (A) Experimental timeline and schematic representation of CAT–Ce6 NCs for in vivo treatment of MRSA-infected subcutaneous abscess. (B) Agar plate images of bacterial culture extracted from abscess tissues of mice in different groups at day 6, and the corresponding quantitative data of bacterial colonies. Data are presented as the mean ± SD (*n* = 3 independent experiments). (C) Representative photographs of infected abscess undergoing different treatments and simulated abscess traces. Scale bar, 1 mm. (D) Quantitative analysis of the infected abscess area for each group in the whole treatment. Data are presented as the mean ± SD (*n* = 5 for each group). (E) H&E staining of abscess tissues in different treatment groups at day 12. Scale bar (2.0×), 500 μm. Scale bar (20×), 50 μm. **P < 0.01 and ***P < 0.001.

Subcutaneous abscess, a representative bacterial infection with severe hypoxic environment and highly expressed H_2_O_2_ in pustules, has become a substantial health concern, affecting across all ages and impacting approximately 8 million people in China [49]. Traditional therapeutic modality, such as puncture and antibiotic administration, often fails to fully eradicate drug-resistant bacteria and even leads to wound enlargement or disease recurrence [50,51]. To assess the therapeutic effect of CAT–Ce6 NCs for in vivo catalysis-augmented PDT of subcutaneous bacterial abscess, an MRSA-infected model of subcutaneous abscess was first established by subcutaneously injecting MRSA into the dorsum of mice. The infected mice were then randomly divided into 5 groups and treated 1 d after MRSA inoculation via subcutaneous injection of PBS, Ce6, CAT, CAT-PVP, and CAT–Ce6 NCs and immediate laser irradiation (Fig. 7A). After 5 d of treatment, bacterial suspensions extracted from infected abscess site were spread on solid agar plates to analyze bacterial burden under different treatments. As shown in Fig. 7B, a considerable number of bacteria were found in the treatment group of Ce6, which was comparable to that of PBS (control), CAT, and CAT-PVP. This phenomenon indicated that Ce6-based traditional PDT was indeed incapable of effective photodynamic bacterial elimination due to the hypoxic pathological microenvironment of subcutaneous abscess. In sharp contrast, bacterial amounts in the treatment group of CAT–Ce6 NCs were substantially reduced as expected, demonstrating that CAT–Ce6 NCs could rapidly eradicate invasive MRSA in hypoxic abscess via catalysis-augmented PDT.

The healing of MRSA-infected abscess under different treatments was monitored in real time. Figure 7C shows representative photographs of MRSA-infected subcutaneous abscess within 11 d of treatment in the 4 groups, and the corresponding graphical representations of the quantitative measurement of abscess areas are presented in Fig. 7D. It was found that the mice treated with CAT–Ce6 NCs showed much faster scarring and small abscess areas compared to the other 4 groups during the entire treatment. The scars of mice almost disappeared in the treatment group of CAT–Ce6 NCs with an abscess healing rate of 95.5% after 11 d of treatment, showing greatly accelerated recovery and healing process of abscess. The healing of mice abscess was further evaluated by histological analysis after 11 d of treatment. Hematoxylin and eosin (H&E) staining results showed that the abscess tissues of mice in the groups of PBS, Ce6, CAT, and CAT-PVP were still in an inflammation phase with severe inflammatory cells and thickening of the epidermis (Fig. 7E). By contrast, the tissue biopsy section treated by CAT–Ce6 NCs exhibited normal skin morphological features with intact epidermis, dermis, blood vessels, and hair follicles. Collectively, by eliminating drug-resistant bacteria in hypoxic pathological microenvironment and accelerating the regeneration and repair of skin tissue, CAT–Ce6 NCs enabled effective in vivo treatment of infected subcutaneous abscess via catalysis-augmented PDT.

## Discussion

By utilizing enzymes to improve the bioavailability of PS and remodel pathological microenvironment via in situ biocatalysis, enzyme–PS conjugates can substantially boost the efficiency of traditional PDT, holding considerable promise for clinical treatment of cancer and infectious diseases. Nowadays, enzyme–PS conjugates are usually prepared by covalent coupling involving complicated chemical synthesis and purification processes in which organic solvent and toxic reagents used would impair the activity of enzymes and result in immunogenicity and inflammation. Physical binding employing noncovalent interactions provides a simple and nondestructive strategy to combine PS with enzymes, but the mechanism and principle of enzyme–PS physical binding remain elusive. In this study, we systematically investigate the physical interactions between enzymes and classic PS of Ce6, and reveal that the positively charged and hydrophobic residues on the surface of enzymes are crucial determinants of Ce6 binding. In addition, a criterion for assessing and predicting the enzyme–Ce6 binding affinity is proposed based on the surface positively charged region area of enzyme. Guided by this criterion, we further construct CAT–Ce6 NCs with superior stability and robust photodynamic antimicrobial capability in hypoxic condition via physical binding. In vivo experiments demonstrate that CAT–Ce6 NCs enable hypoxia pathological microenvironment remodeling and bacteria elimination, realizing effective catalysis-augmented PDT of MRSA-infected subcutaneous abscess. This study establishes a theoretical framework to guide the design of physically bonded enzyme–PS NCs and facilitate catalysis-augmented PDT.

## Materials and Methods

### Materials

Proteinase K, Lys, GOx, CAT, and HSA were purchased from Shanghai Yuanye Bio-Technology Co. Ltd. Dimethyl sulfoxide (DMSO), Ce6, vancomycin, singlet oxygen sensor green (SOSG), 2′,7′-dichlorodihydrofluorescein diacetate (DCFH-DA), cell counting kit-8 (CCK-8), and PVP (*M*_w_ = 10 kDa) were obtained from Sigma-Aldrich. Sensor CM5 chip and amine coupling kit were purchased from Cytiva. H_2_O_2_ assay kit was obtained from Shanghai Beyotime Biotechnology Co. Ltd. BCA protein assay kit was purchased from Nanjing Jiancheng Bioengineering Research Institute. Live/dead bacterial viability kit was purchased from Thermo Fisher Scientific. HUVECs, mouse embryonic fibroblast cells (NIH/3T3), MRSA, and *E. coli* were obtained from the American Type Culture Collection (ATCC). All other chemicals were obtained from Adamas-beta and used without further purification. Deionized (DI) water (Millipore Milli-Q grade, 18.2 MΩ) was used in all the experiments.

### MD simulation of the physical binding of enzyme–Ce6

In all simulations, one enzyme was centered in a cubic simulation box with a size of 16 × 16 × 16 nm^3^. The box was large enough for even the biggest enzyme in this study (CAT) so that the minimum distance between the enzyme and the edge of the box was still larger than 2 nm. Twenty Ce6 molecules were then added to the simulation box, with the criterion that the center of mass of each Ce6 molecule was at least 2 nm away from the enzyme. The way we set up our simulation box ensured a comparable concentration of Ce6 among all the simulated systems. The complex of enzyme and Ce6 was then solvated in TIP3P water [52], and Na^+^ counterions were added to the solvent to neutralize the net charge of the complex. All MD simulations were conducted using the GROMACS2019 package. The GROMOS united-atom force field was applied to describe the interactions in the simulation systems. The topology of Ce6 was built by the Automated Topology Builder (ATB). The leap-frog algorithm was used to integrate Newton’s equations of motion, and all the bonds with H atoms were constrained by the LINCS algorithm [53] to eliminate the fast vibrations so that the integration step could be longer, which was 2 fs in this case. The cutoffs of the short-range electrostatics and van der Waals interactions were both set to 1.2 nm, and the long-range electrostatic interaction was treated with the particle mesh Ewald (PME) algorithm [54]. Before the production simulation, all the systems were prepared in a same protocol: an energy minimization followed by 100-ps equilibration under the canonical ensemble (NVT ensemble) and another 100-ps equilibration under the isothermal-isobaric ensemble (NPT ensemble) (300 K and 1 atm). Position restraints were applied to the protein heavy atoms during equilibration. The V-rescale algorithm and isotropic Berendsen algorithm were used for temperature coupling and pressure coupling [55,56], respectively. After equilibration, 3 independent trajectories of 300 ns were generated for each simulation system.

### Determination of the binding affinity of enzyme–Ce6

SPR analysis was conducted to measure the equilibrium dissociation constant (*K*_D_) of Ce6 to enzymes to evaluate the binding affinity of enzyme–Ce6. In detail, enzymes were first immobilized on the Sensor Chip CM5 using an amine coupling kit, with a blank channel serving as the control. Different concentrations of Ce6 solution were then applied to flow slowly through the control and immobilized enzyme channels at a flow rate of 20 μl/min. After each cycle, the chip surface was regenerated with DMSO at a flow rate of 20 μl/min for 30 s. HBS-EP^+^ (Hepes-buffered saline with EDTA and surfactant P20, pH 7.4) was used as the running buffer in all experiments. All data were finally fitted and processed using the Biacore T200 evaluation software to calculate the value of *K*_D_.

### Preparation of CAT–Ce6 NCs

CAT (1 mg) and PVP (6.7 mg) were dissolved in 300 μl of DI water and 1 ml of PBS (0.01 M, pH 7.4), respectively. Additionally, 1 mg of Ce6 was dispersed in 250 μl of 0.1% NaOH solution. The prepared Ce6 solution (125 μl) and the PVP solution (75 μl) were added dropwise to the CAT solution (300 μl) under stirring. After 10-min reaction, CAT–Ce6 NCs were obtained and purified through multiple centrifugations (4,000*g*, 15 min) using an ultrafiltration tube (molecular weight cutoff = 50 kDa).

### CAT catalytic activity of CAT–Ce6 NCs

The CAT activity of CAT–Ce6 NCs was assessed by measuring the dissolved O_2_ levels in the presence of H_2_O_2_. In brief, free CAT (10 μg/ml) and CAT–Ce6 NCs (equivalent to 10 μg/ml of CAT) were mixed with H_2_O_2_ solution (1 × 10^−4^ M). The content of dissolved O_2_ in the mixture of CAT–Ce6 NCs and H_2_O_2_ was subsequently measured and compared with that of the mixture of CAT and H_2_O_2_, using a portable dissolved oxygen meter O_2_ probe (JPBJ-608, Shanghai REX Instrument Factory) to indicate their CAT catalytic activities.

### Stability of CAT–Ce6 NCs to resist protease hydrolysis and photobleaching

The stability of CAT–Ce6 NCs to resist protease hydrolysis was evaluated by analyzing their CAT activity in the presence of proteinase K. Briefly, free CAT (100 μg/ml) and CAT–Ce6 NCs (equivalent to 100 μg/ml of CAT) were incubated with proteinase K (20 μg/ml) at 37 °C, respectively. Equal samples were taken at predetermined time points (0, 1, 2, 4, 6, and 12 h), and their CAT activities were tested and compared using the catalase assay kit.

The stability of CAT–Ce6 NCs to resist photobleaching was evaluated by analyzing their Ce6 fluorescence under continuous laser irradiation. In brief, free Ce6 (5 μg/ml) and CAT–Ce6 NCs (equivalent to 5 μg/ml of Ce6) were irradiated by laser (660 nm, 0.8 W/cm^2^) for 30 min. Equal samples were taken at predetermined time points of irradiation (0, 5, 10, 15, 20, 25, and 30 min), and their Ce6 fluorescence was measured and compared.

### Bacteria culture and antimicrobial capability of CAT–Ce6 NCs in hypoxic condition

MRSA (ATCC 33591) and *E. coli* (ATCC 8739) were used in our experiments. The bacteria were cultured in lysogeny broth (LB) medium and harvested at the exponential growth phase before use. For antimicrobial experiments, 10^6^ colony-forming units (CFU) of MRSA or *E. coli* bacteria were first mixed with CAT–Ce6 NCs (1 μg/ml of Ce6) in Ar environment upon laser irradiation (660 nm, 0.8 W/cm^2^, 10 min) with/without H_2_O_2_ (1 × 10^−4^ M) incubation. CFUs were then measured using solid agar plates to assess the in vitro photodynamic antimicrobial effect of CAT–Ce6 NCs in hypoxic condition. Additionally, vancomycin (2 mg/ml) was mixed with 10^6^ CFU of MRSA in Ar environment with/without H_2_O_2_ (1 × 10^−4^ M) incubation as a positive control.

### Live/dead bacterial staining assay

MRSA or *E. coli* bacteria (10^6^ CFU) were first mixed with CAT–Ce6 NCs (1 μg/ml of Ce6) in Ar environment upon laser irradiation (660 nm, 0.8 W/cm^2^, 10 min) with/without H_2_O_2_ (1 × 10^−4^ M) incubation. Then, bacterial samples were incubated with dye solutions containing SYTO 9 and propidium iodide for 30 min in the dark and then imaged using a confocal fluorescence microscopy. Viable bacteria were stained green with SYTO 9, while dead bacteria were stained red with propidium iodide due to the damage of cell wall and membrane.

### Intracellular ROS measurement

The intracellular ROS level of bacteria was measured using the DCFH-DA probe, which was oxidized by ROS to yield the 2′,7′-dichlorofluorescein (DCF) with green fluorescence. Briefly, 10^6^ CFU of MRSA or *E. coli* bacteria were first mixed with CAT–Ce6 NCs (1 μg/ml of Ce6) in Ar environment upon laser irradiation (660 nm, 0.8 W/cm^2^, 10 min) with/without H_2_O_2_ (1 × 10^−4^ M) incubation. Then, bacterial samples were mixed with DCFH-DA probe at 37 °C for 20 min in the dark, and the intracellular ROS level was evaluated using a confocal fluorescence microscope after multiple washes with PBS solution.

### SEM-based bacterial morphological study

MRSA or *E. coli* bacteria (10^6^ CFU) were first mixed with CAT–Ce6 NCs (1 μg/ml of Ce6) in Ar environment upon laser irradiation (660 nm, 0.8 W/cm^2^, 10 min) with/without H_2_O_2_ (1 × 10^−4^ M) incubation. The treated bacterial samples were then fixed by 2.5% glutaraldehyde in the dark for 2 h and then dehydrated with ethanol solutions of increasing concentrations (50%, 70%, 90%, and 100%) for 10 min. After dehydration and drying, bacterial morphology was observed using a cold field-emission scanning electron microscope (JSM-6700F).

### Construction of mouse model of MRSA-induced subcutaneous abscess

Female BALB/c mice (6 to 8 weeks old) were purchased from Jinan Pengyue Laboratory Animal Breeding Corporation and allowed to acclimate to the laboratory for 1 week before the experiment. All animal experiments were conducted in accordance with the protocols approved by the Laboratory Animal Center of Shandong University. All the mice were depilated and sterilized before establishing the model. After anesthesia, 50 μl of MRSA suspension (10^8^ CFU/ml) was injected into the subcutaneous layer of mice to construct the model of MRSA-induced subcutaneous abscess.

### In vivo catalysis-augmented PDT of MRSA-infected subcutaneous abscess

To perform in vivo treatment, MRSA-infected mice were administrated with CAT–Ce6 NCs 1 d after bacterial inoculation via subcutaneous injection. Four treatment groups (5 mice per group) were divided including PBS, Ce6 (100 μg/ml), CAT (840 μg/ml), CAT-PVP (840 μg/ml of CAT), and CAT–Ce6 NCs (containing 100 μg/ml of Ce6 and 840 μg/ml of CAT). Laser irradiation (660 nm, 0.8 W/cm^2^, 10 min) was carried out after injection for the 4 treatment groups. The abscesses of mice in the 4 treatment groups were photographed every 2 d, and the abscess areas were calculated using ImageJ software. After 5 d of treatment, bacterial suspensions extracted from the infected abscess sites were spread on solid agar plates to analyze bacterial burden under different treatments. After 11 d of treatment, all mice were euthanized, and their abscess tissues of skin were collected for histological H&E staining.

## Data Availability

The data that support the findings of this study are available within the article and its Supplementary Materials. Raw data are available from the corresponding authors on reasonable request.

## Supplementary Materials

Supplementary 1 Experimental Section Figs. S1 to S35 Tables S1 to S6
