# Supplementary material for: Deciphering the Physical Binding Mechanism of Enzyme–Photosensitizer Facilitates Catalysis-Augmented Photodynamic Therapy
Source: Research (Wash D C). 2025 Jun 3;8:0732. doi: 10.34133/research.0732 (PMC12133102; doi:10.34133/research.0732)

**Supporting Information**

**Deciphering the Physical Binding Mechanism of Enzyme-Photosensitizer Facilitates Catalysis-Augmented Photodynamic Therapy**

Bingqing Jia^1,§^, Yang Liu^1,§^, Xudong Geng^1^, Yuezheng Li^1^, Chengmei Zhang^2^, Yuanyuan Qu^1^, Xiangdong Liu^1^, Mingwen Zhao^1^, Yanmei Yang^3^*, Weifeng Li^1^, Yong-Qiang Li^1^*

^1^ Institute of Advanced Interdisciplinary Science, School of Physics, Shandong University, Jinan 250100, China.

^2^ Laboratory Animal Center of Shandong University, Jinan 250012, China.

^3^ College of Chemistry, Chemical Engineering and Materials Science, Collaborative Innovation Centre of Functionalized Probes for Chemical Imaging in Universities of Shandong, Key Laboratory of Molecular and Nano Probes, Ministry of Education, Shandong Normal University, Jinan, 250014, China.

^§^ These authors contributed equally to this work.

1. Experimental

***Catalytic Activity of CAT-Ce6 NCs under Varying pH and Temperature Conditions***

The catalytic activity of CAT-Ce6 NCs was systematically evaluated by analyzing CAT activity under varying pH and temperature conditions. Briefly, free CAT (100 µg/mL) and CAT-Ce6 NCs (equivalent to 100 µg/mL of CAT) was incubated with the buffer solutions with varied pH (2, 3, 4, 5, 6, 7, 8, 9, 10, 11) at 37 °C, respectively. Subsequently, free CAT (100 µg/mL) and CAT-Ce6 NCs (equivalent to 100 µg/mL of CAT) was added into 3 mL buffer solution (pH = 7) in a water bath at different temperatures (0, 10, 20, 30, 40, 50, 60 ℃) for 10 min. Equal samples were taken, and their CAT activities were tested and compared using the catalase assay kit.

***In Vitro Biocompatibility of CAT-Ce6 NCs***

The *in vitro* biocompatibility of CAT-Ce6 NCs was determined by MTT assay using human umbilical vein endothelial cells (HUVEC) and mouse embryonic fibroblast cells (NIH/3T3). In brief, HUVEC or NIH/3T3 cells were seeded into a 96-well plate (8000-10000 cells/well) and cultured overnight. Then the cells were treated by CAT-Ce6 NCs with different concentrations (0, 50, 100, and 150 µg/mL of Ce6). After 24 and 48 h of culture, MTT reagent was added and the cell viability was evaluated with a microplate reader, respectively.

***In Vivo Biocompatibility of CAT-Ce6 NCs***

Organ pathological examination and blood routine assay were conducted to evaluate the *in vivo* biosafety of CAT-Ce6 NCs. In brief, CAT-Ce6 NCs (containing 100 µg/mL of Ce6 and 840 µg/mL of CAT) was subcutaneously injected into healthy mice. Major organs and blood samples were collected on the 12^th^ day of CAT-Ce6 NCs post-injection. HE staining of organs, blood routine assay were performed to demonstrate the *in vivo* biocompatibility of CAT-Ce6 NCs. Organ pathological examination and blood routine assay of healthy mice injected with PBS, CAT, Ce6, CAT-PVP, and CAT-Ce6 NCs were used as the control.

*Optimization of Photodynamic Parameters for CAT-Ce6 NCs*

The photodynamic parameters were systematically optimized by assessing the antibacterial activity of CAT-Ce6 NCs under varying wavelengths, power densities, and irradiation durations. In brief, 10^6^ CFU of MRSA were co-incubated with CAT-Ce6 NCs (1 µg/mL of Ce6) and irradiated for 10 minutes using distinct laser wavelengths (660, 730, 808, and 1064 nm). The photodynamic efficacy was assessed by enumerating colony-forming units (CFUs) on solid agar plates after treatment. Subsequently, the laser power density (0, 0.2, 0.4, 0.6, 0.8 and 1 W/cm^2^) and irradiation duration (0, 2, 4, 6, 8, and 10 min) were systematically varied using the same experimental protocol to determine the optimal photodynamic parameters.

*Statistical Analysis*

All data in this work were shown as means ± standard deviation (SD). Unless otherwise mentioned, all statistical analyses were conducted by using GraphPad Prism 8.0. The difference between two groups was performed using Student’s t test, and multiple group comparisons were analyzed by one-way two-sided analysis of variance (ANOVA). ^*^*P* < 0.05, ^**^*P* < 0.01, and ^***^*P* < 0.001 were considered statistically significant. All experiments were repeated independently more than three times.

2. Supporting figures


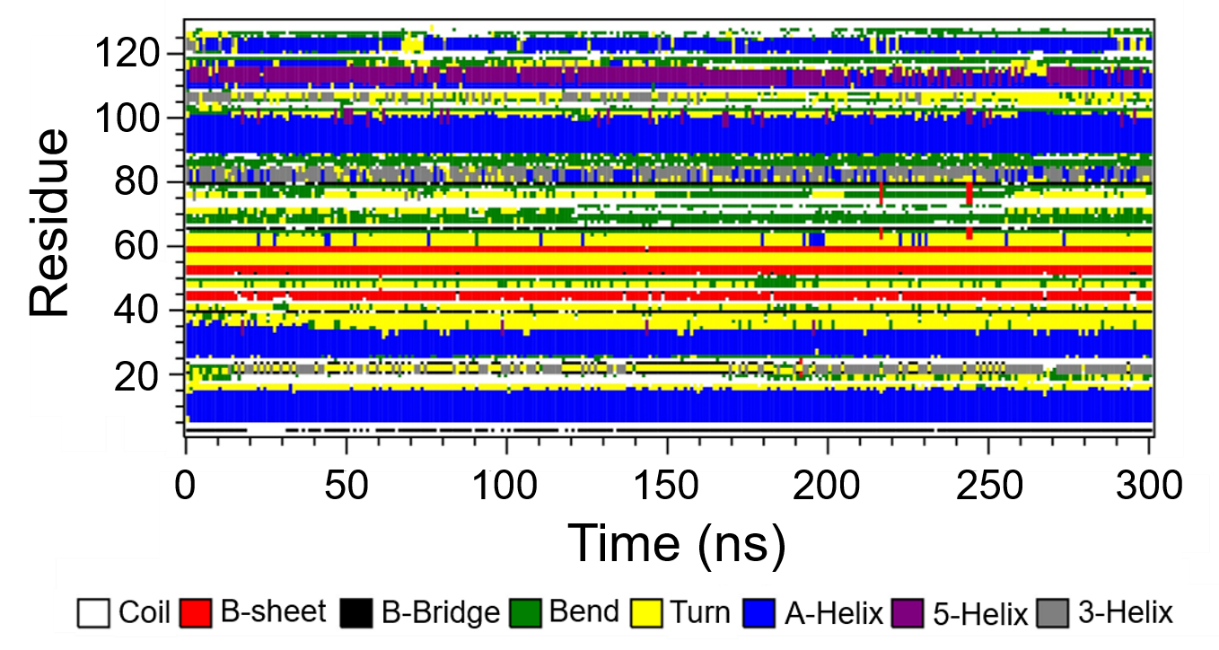


Figure S1. Time evolution of the secondary structure of Lys in Lys-Ce6 system during simulation.


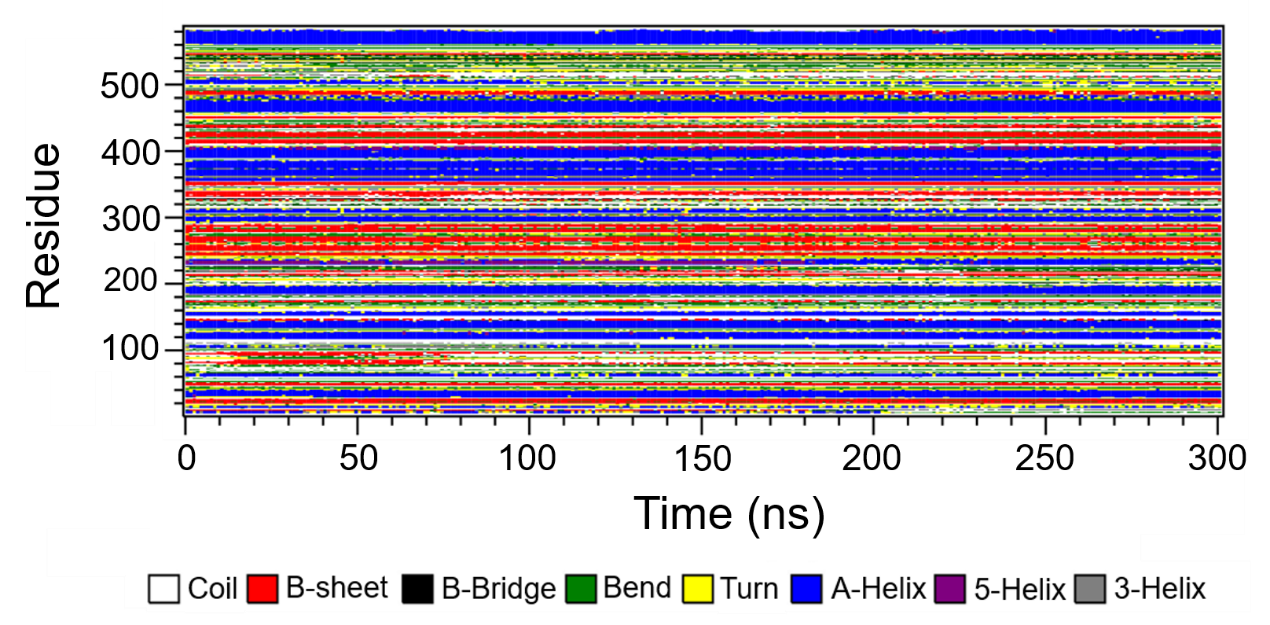


Figure S2. Time evolution of the secondary structure of GOx in GOx-Ce6 system during simulation.


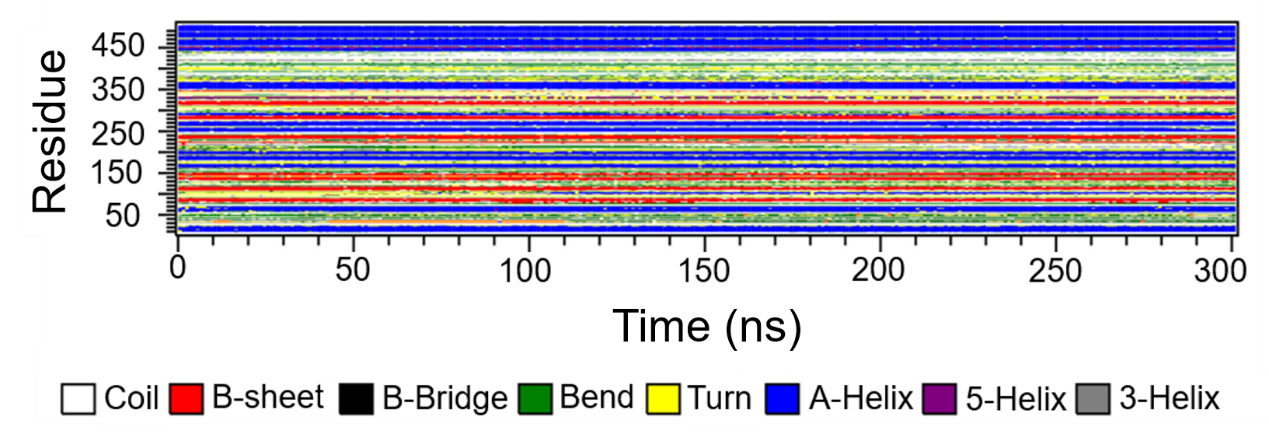


Figure S3. Time evolution of the secondary structure of CAT in CAT-Ce6 system during simulation.


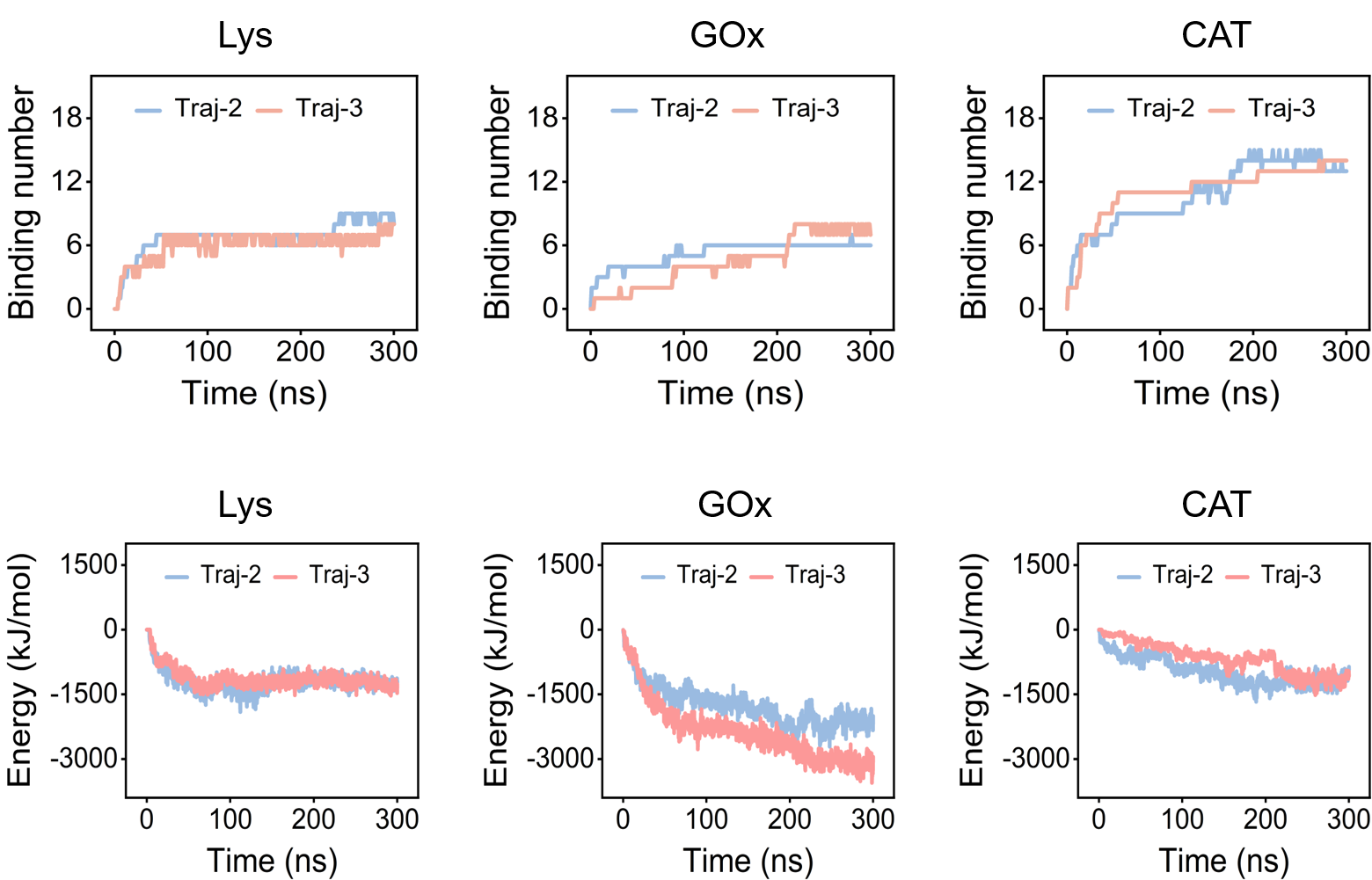


**Figure S4**. The contact numbers and binding energies of Ce6 to Lys, GOx and CAT, respectively.


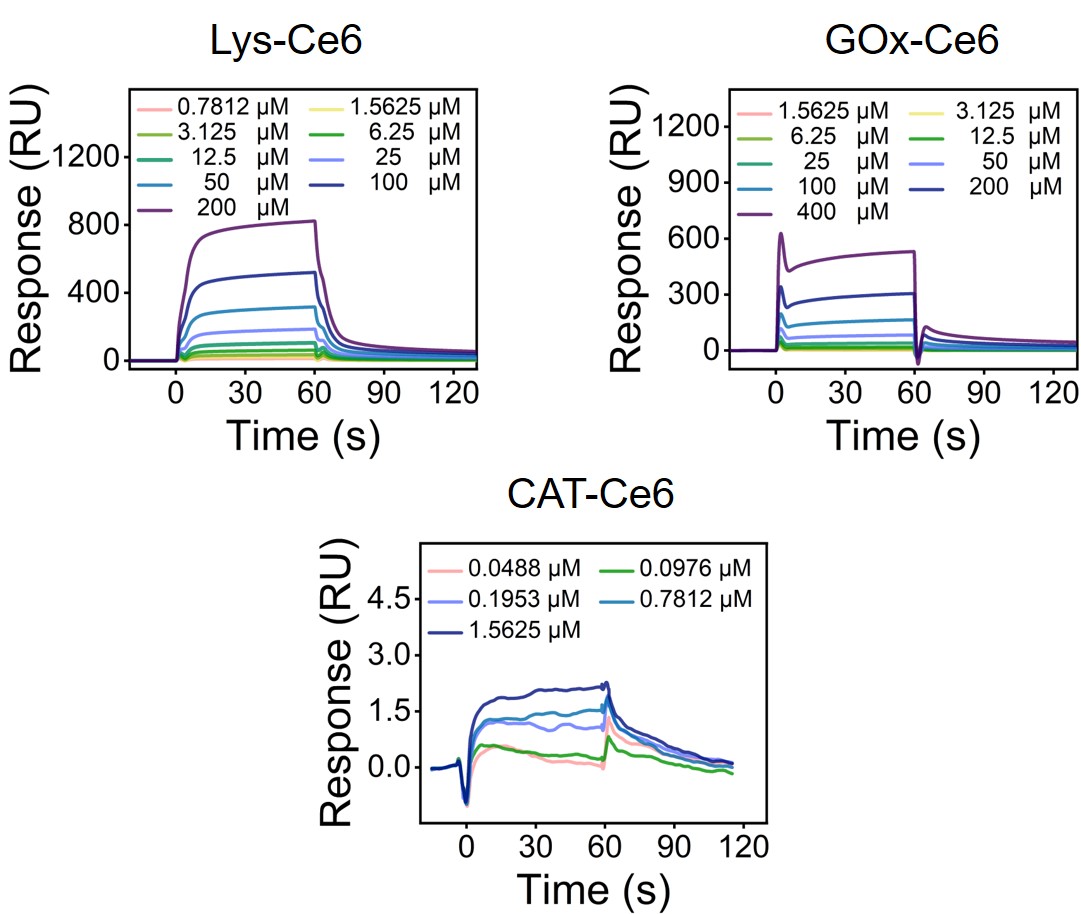


Figure S5. Biocore kinetics assay sensorgrams of Ce6 to Lys, GOx, and CAT.


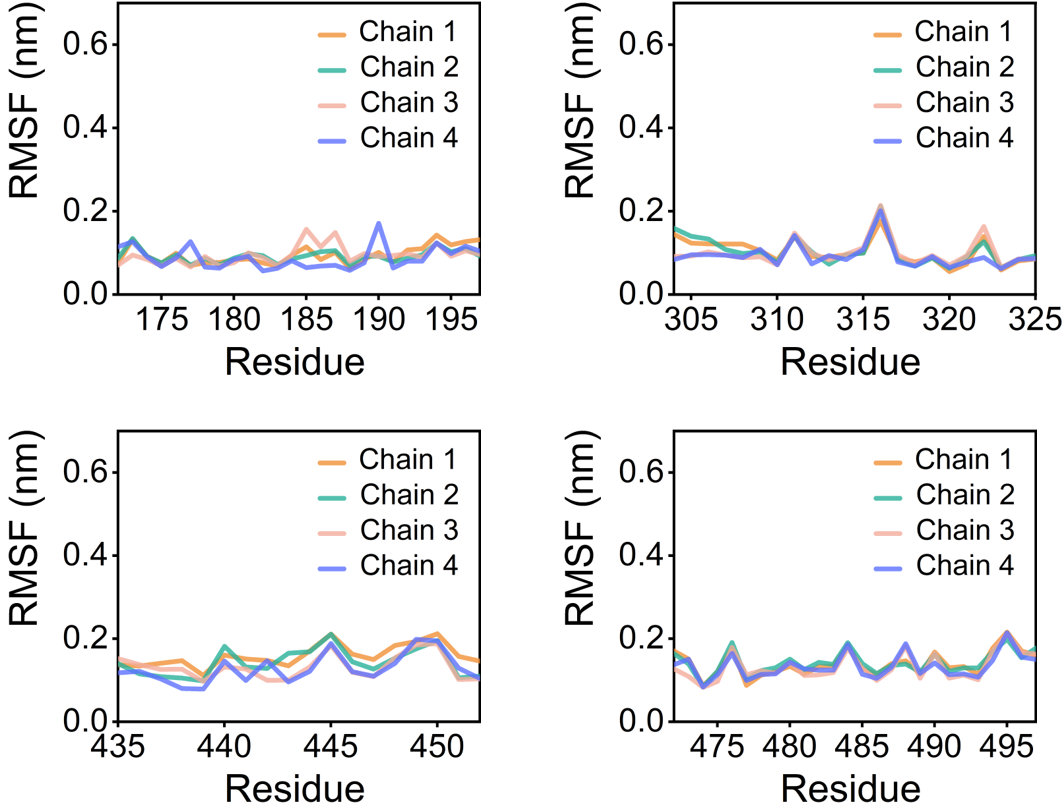


Figure S6. The RMSF of the residues within the binding pocket in CAT-Ce6 system.


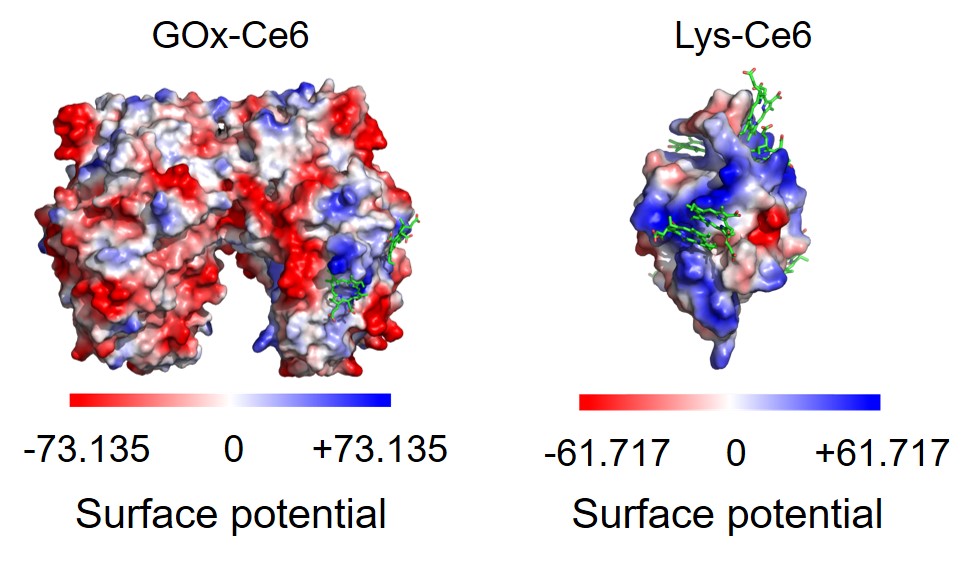


Figure S7. The surface electrostatic potential maps of GOx-Ce6 and Lys-Ce6. The red and blue color represents the negatively charged region and positively charged region, respectively.


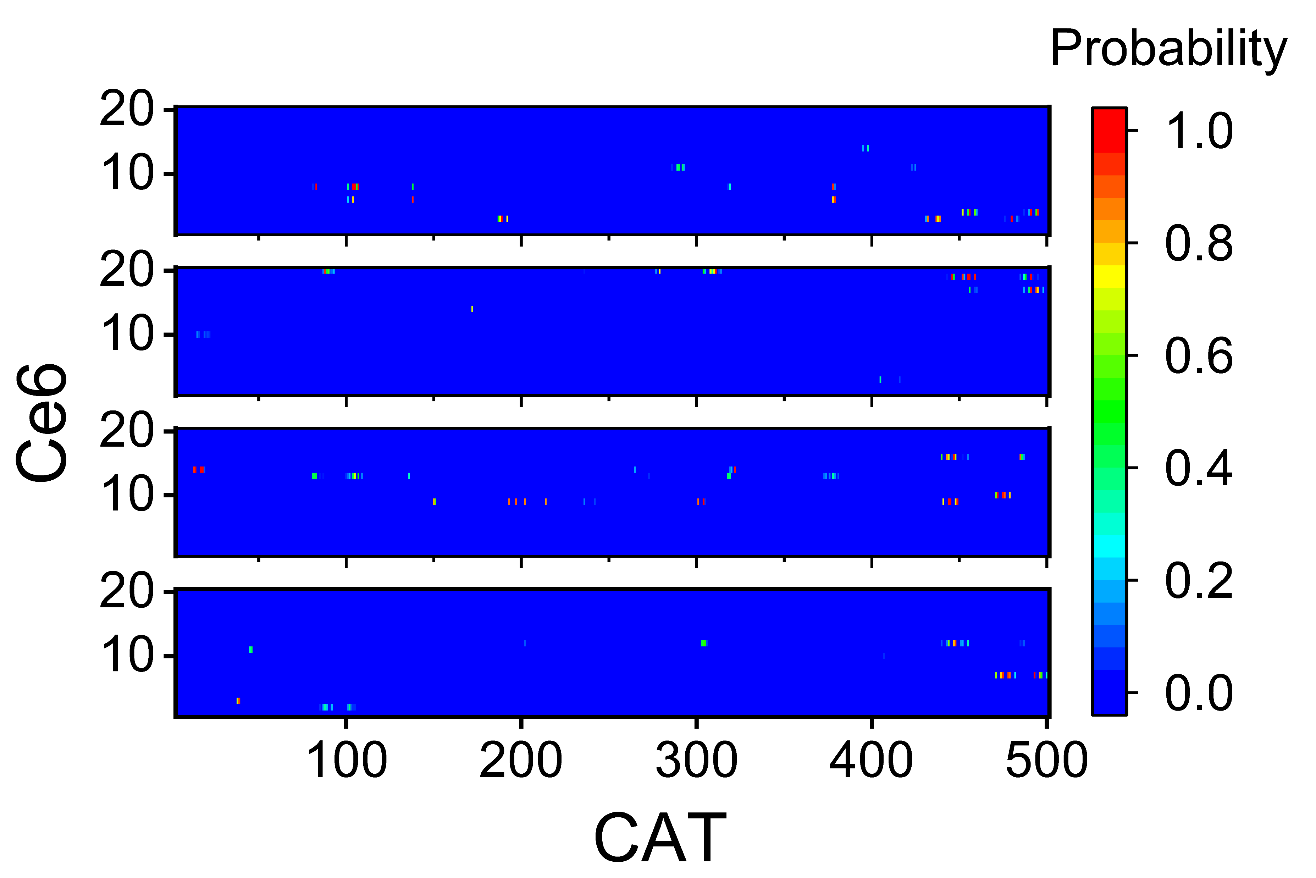


Figure S8. Contact probability of each residue of CAT to Ce6.


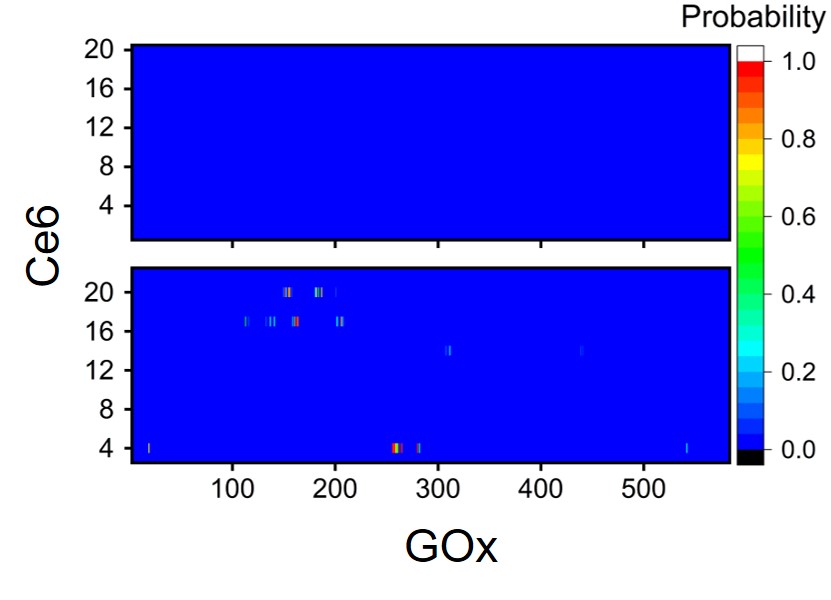


Figure S9. Contact probability of each residue of GOx to Ce6.


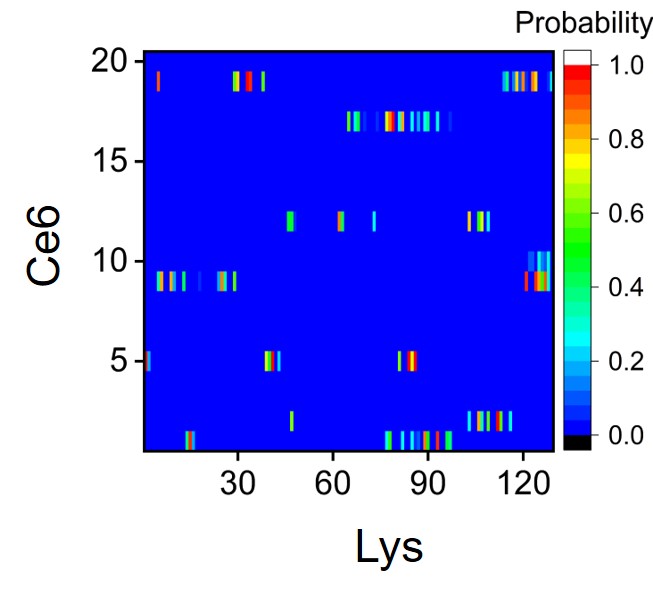


Figure S10. Contact probability of each residue of Lys to Ce6.


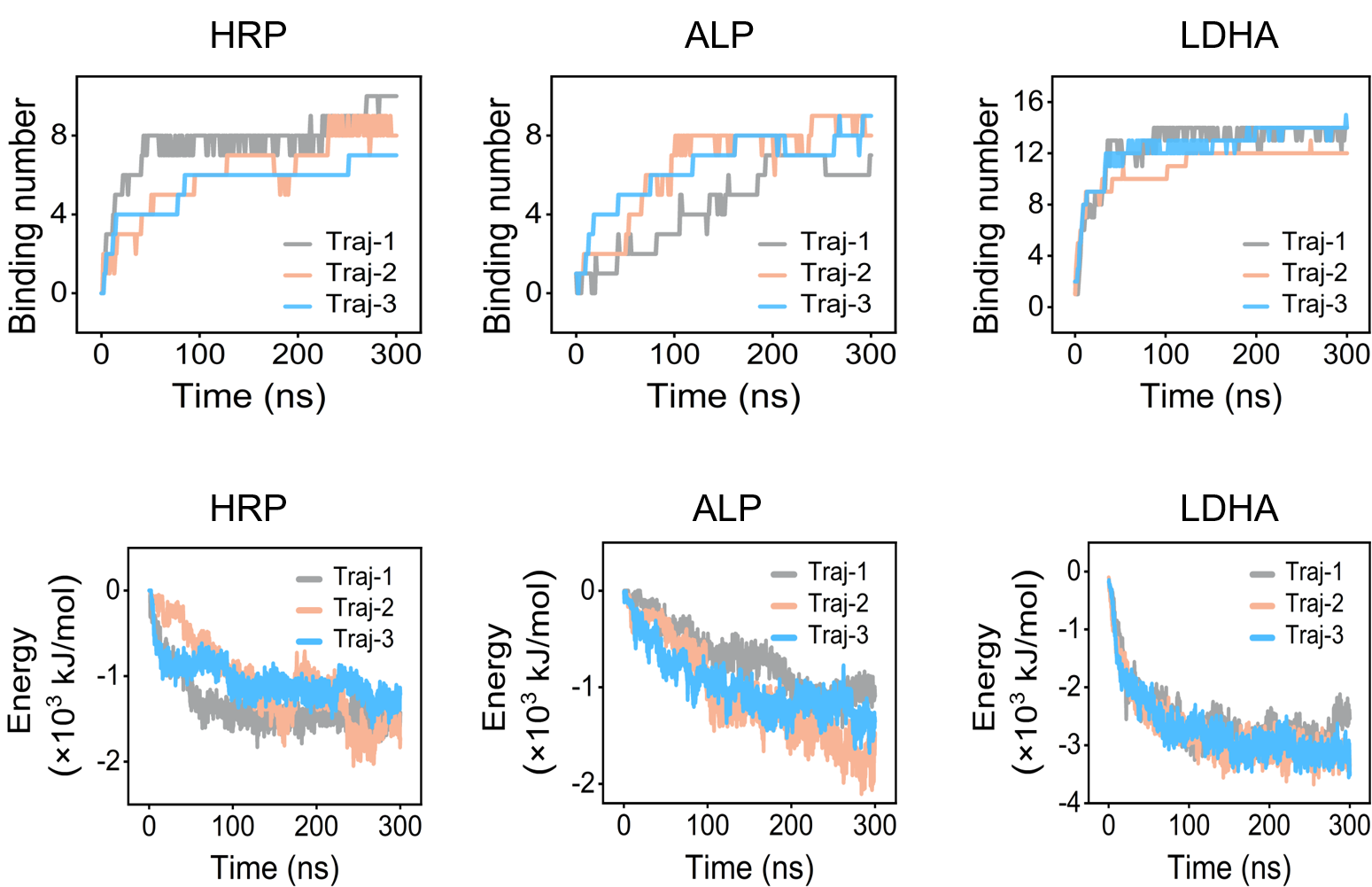


Figure S11. The contact numbers and binding energies of Ce6 to HRP, ALP and LDHA, respectively.


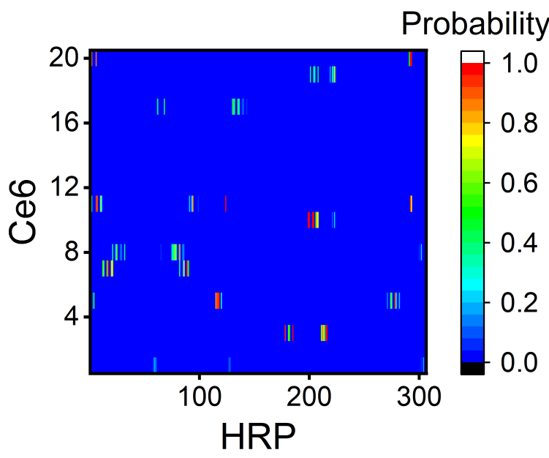


Figure S12. Contact probability of each residue of HRP to Ce6.


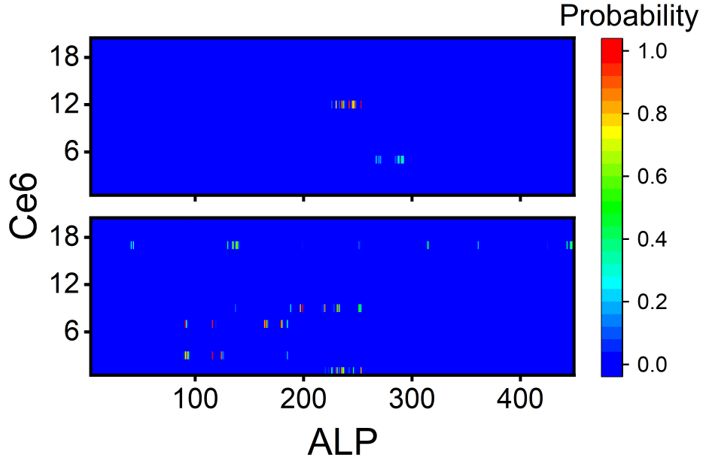


Figure S13. Contact probability of each residue of ALP to Ce6.


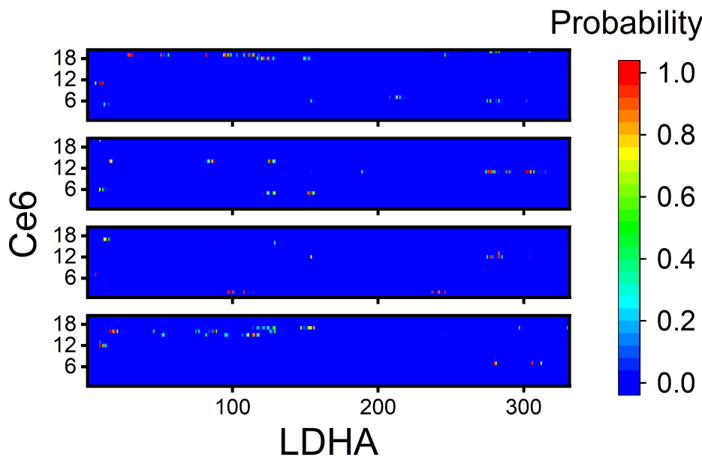


Figure S14. Contact probability of each residue of LDHA to Ce6.


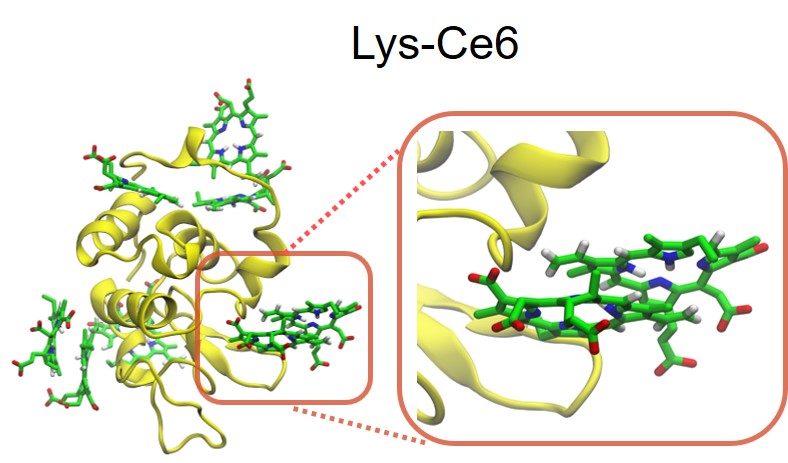


Figure S15. The binding conformation of Lys-Ce6 illustrates the event of Ce6 molecules stacking via π-π interaction.


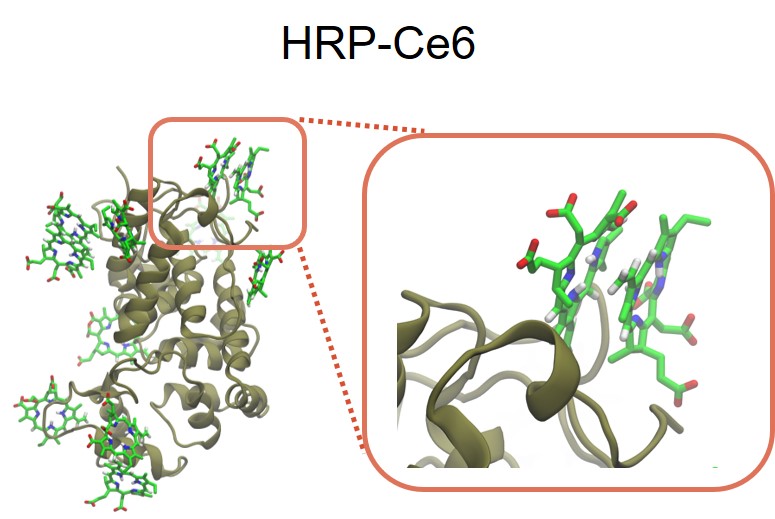


Figure S16. The binding conformation of HRP-Ce6 illustrates the event of Ce6 molecules stacking via π-π interaction.


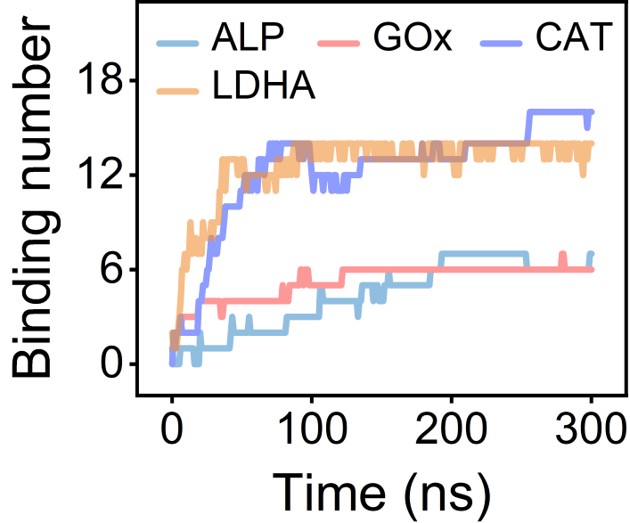


Figure S17. Time evolution of the contact number of Ce6 to ALP, GOx, CAT, and LDHA, respectively, during MD simulation.


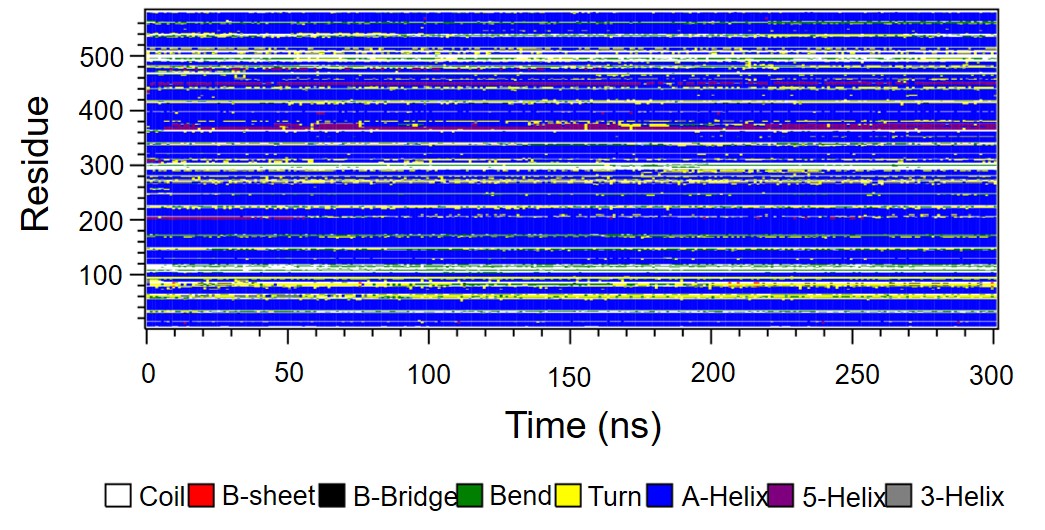


Figure S18. Time evolution of the secondary structure of HSA in HSA-Ce6 system during simulation.


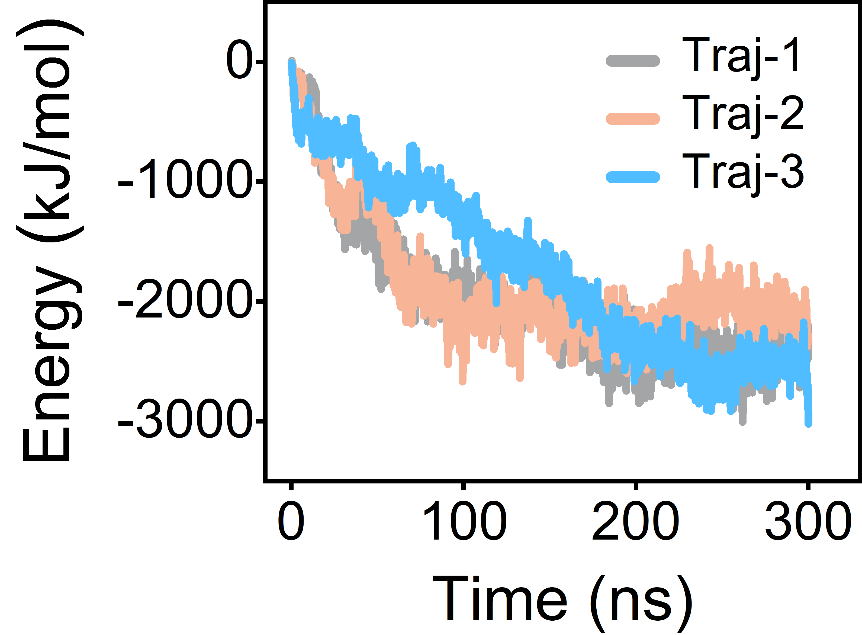


Figure S19. Time evolution of interaction energy between HSA and Ce6 during simulation.


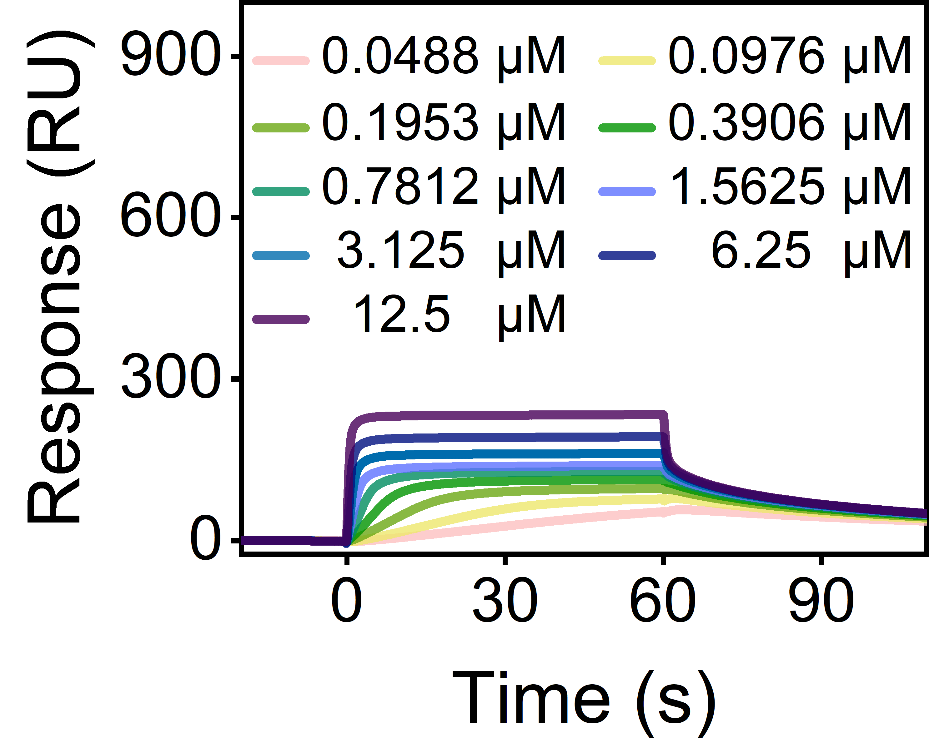


Figure S20. Biocore kinetics assay sensorgrams of Ce6 to HSA.


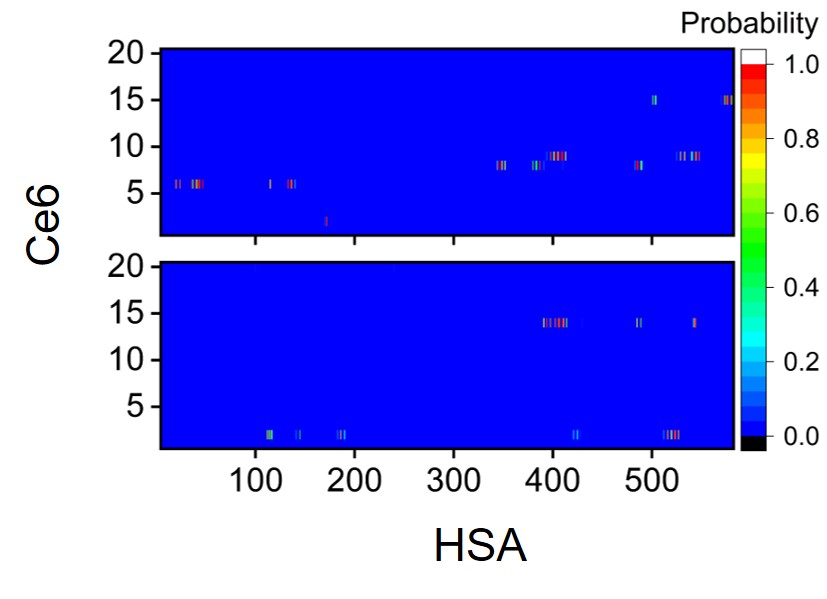


**Figure S21.** Contact probability of each residue of HSA to Ce6.


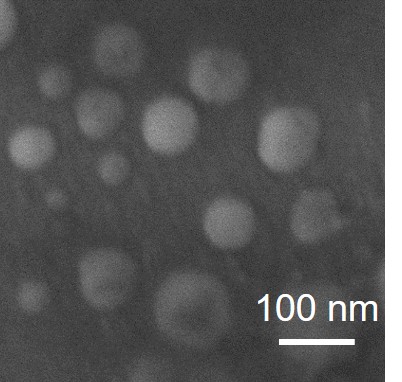


**Figure S22.** SEM image of CAT-Ce6 NCs.


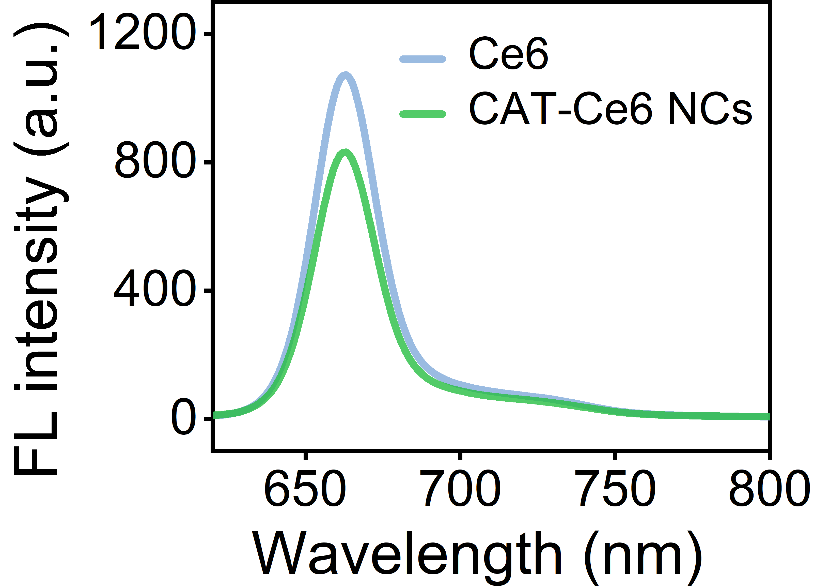


**Figure S23.** Fluorescence spectra of CAT-Ce6 NCs and free Ce6.


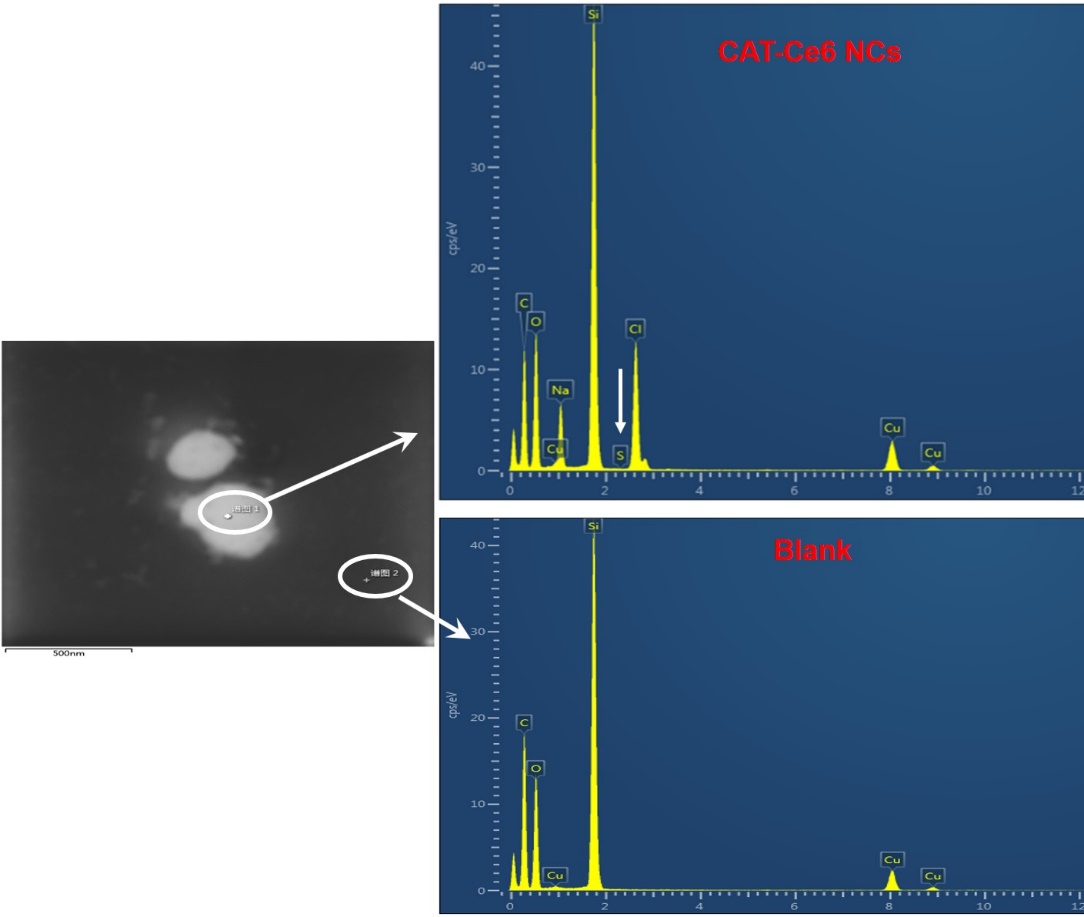


Figure S24. Representative STEM image and EDS mapping of CAT-Ce6 NCs.


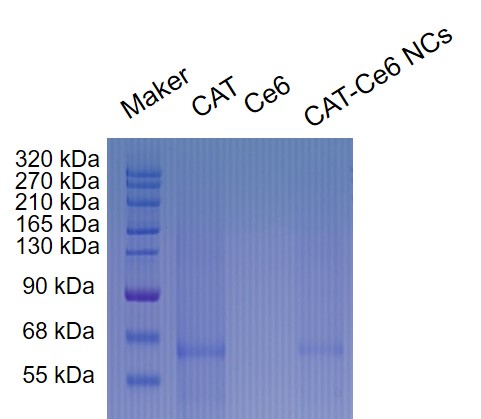


**Figure S25.** SDS-PAGE of CAT, Ce6, and CAT-Ce6 NCs.


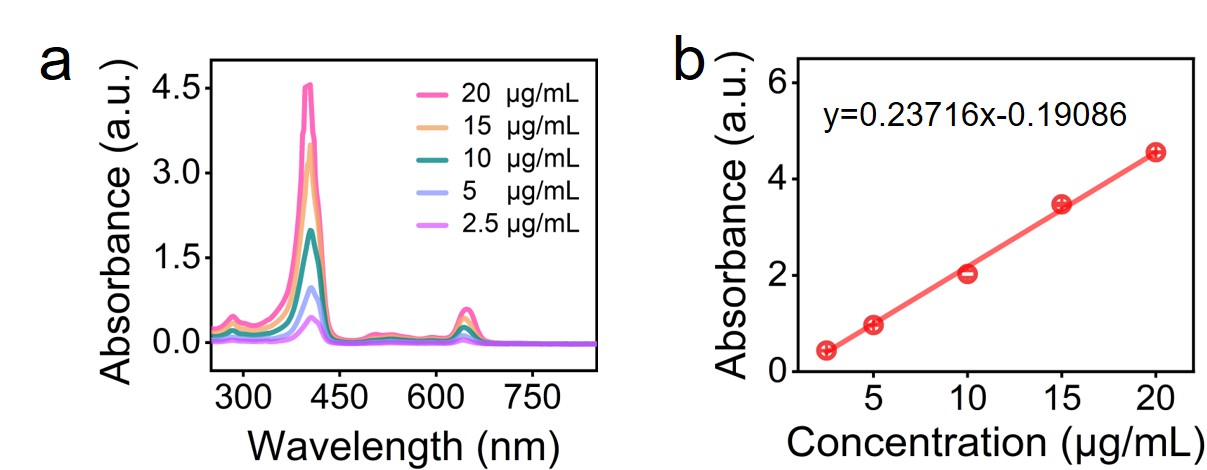


Figure S26. (a) Absorption spectra of Ce6 with various concentrations ranged from 2.5 to 20 µg/mL. (b) The linear calibration curve between Ce6 concentration and its absorbance.


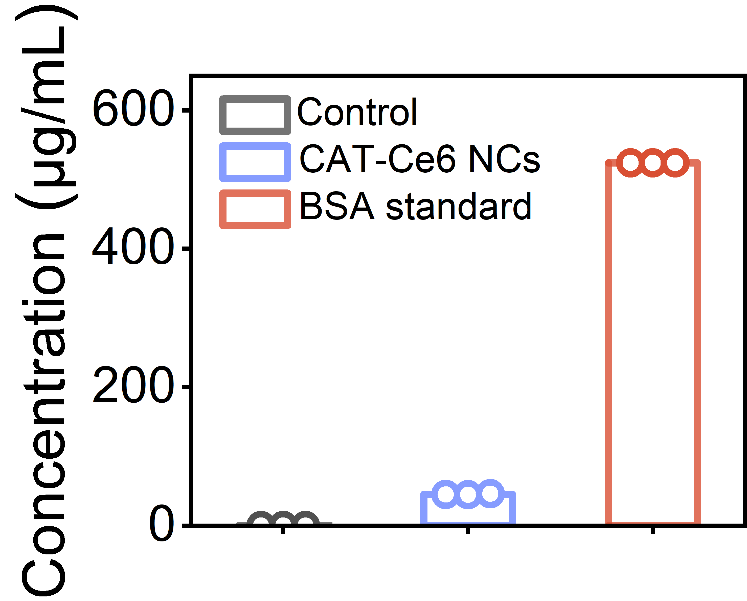


Figure S27. Quantitative CAT concentration in CAT-Ce6 NCs calculated by BCA assay (BSA standard: 524 μg/mL). The values of CAT concentration represent the mean of three independent experiments, and the error bars indicate the SD from the mean.


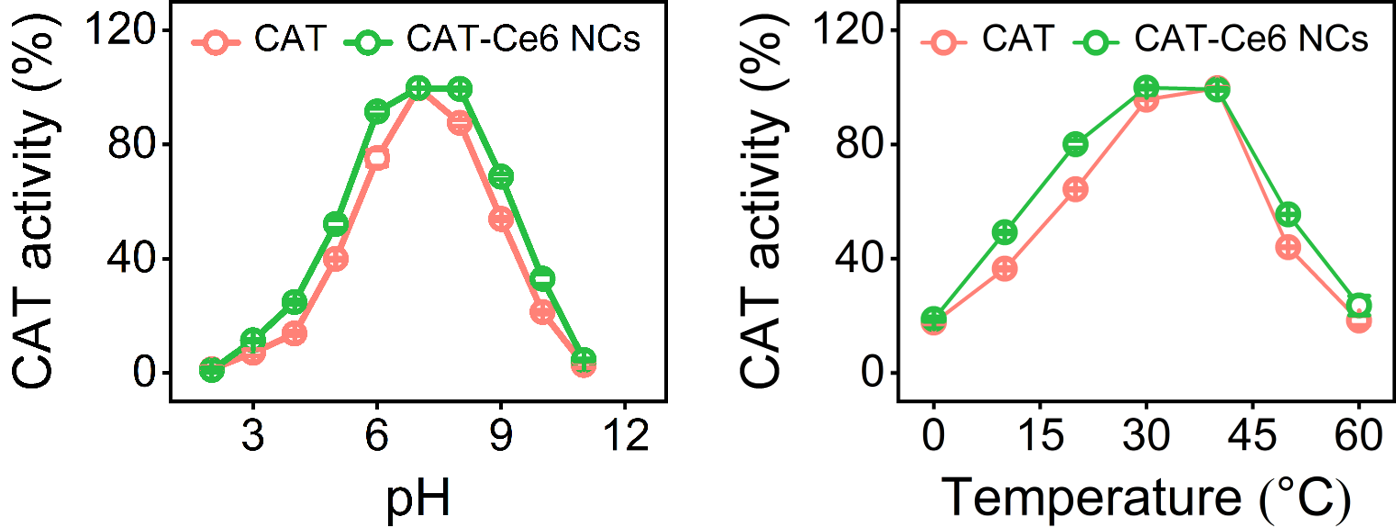


Figure S28. The effects of pH and temperature on the catalytic activity of CAT and CAT-Ce6 NCs. Data are represented as mean ± SD (n = 3).


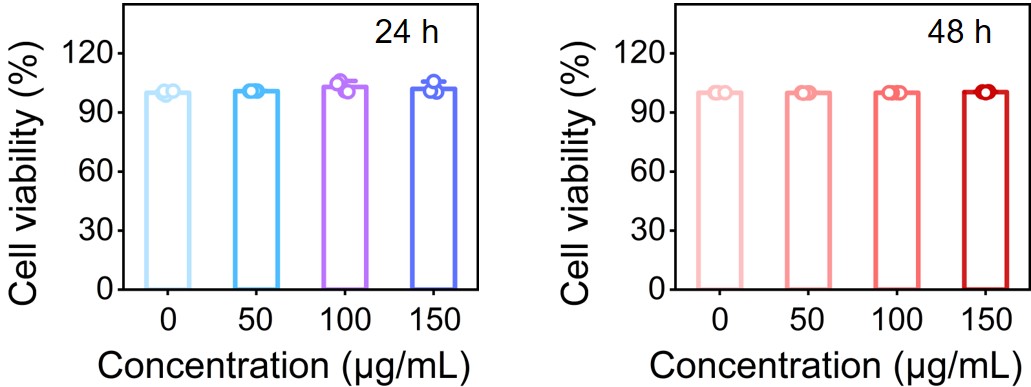


Figure S29. The viability of NIH/3T3 cells after incubation with different concentrations of CAT-Ce6 NPs for 24 and 48 h, respectively. Data are represented as mean ± SD (n = 3).


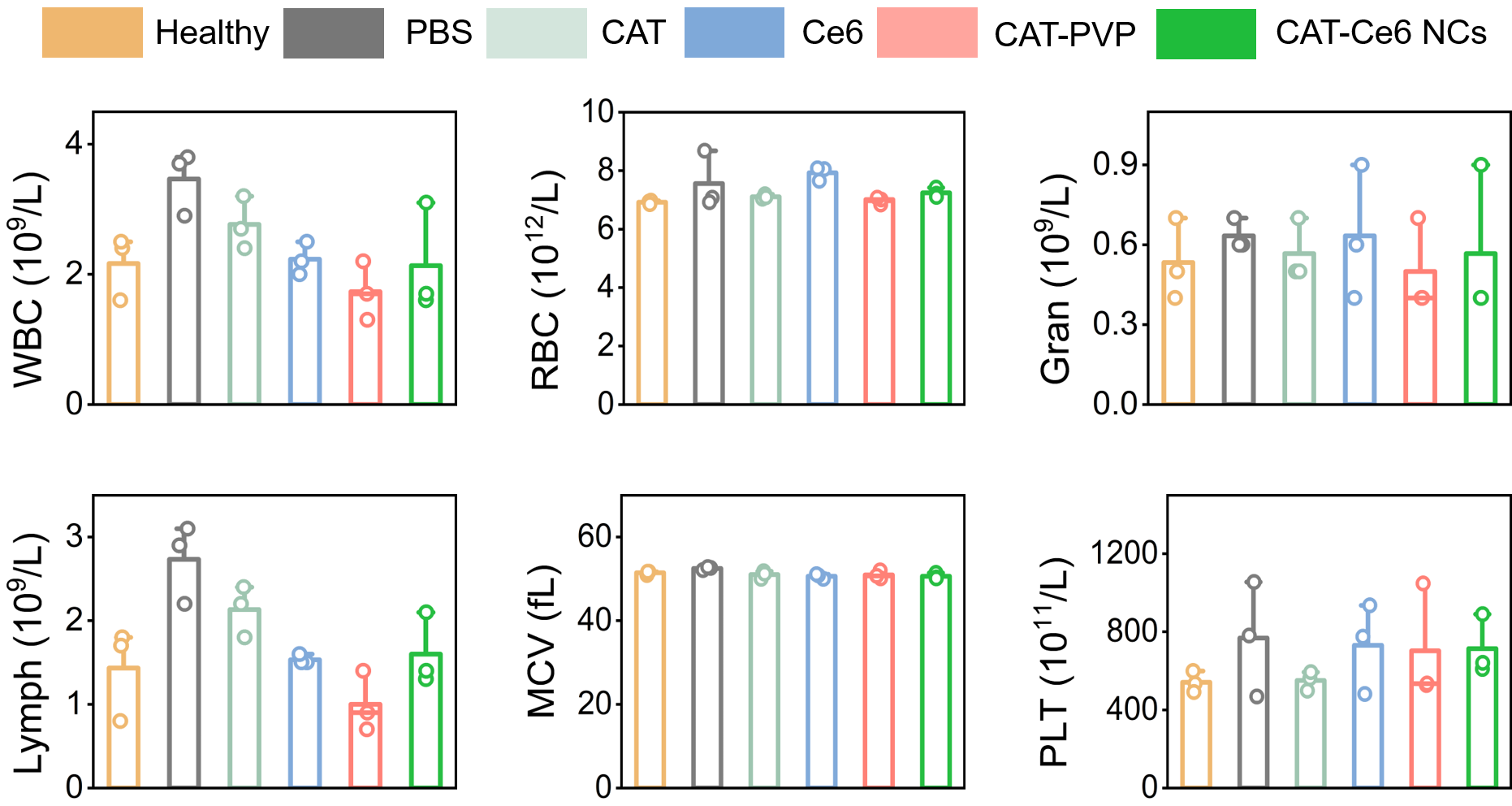


Figure S30. Blood routine examination of mouse subcutaneously injected with PBS, CAT, Ce6, CAT-PVP, and CAT-Ce6 NCs for 12 days, respectively. WBC stands for white blood cell, RBC stands for red blood cell, Gran stands for granulocyte, Lymph stands for lymphocyte, MCV stands for mean corpuscular volume, and PLT stands for platelet.


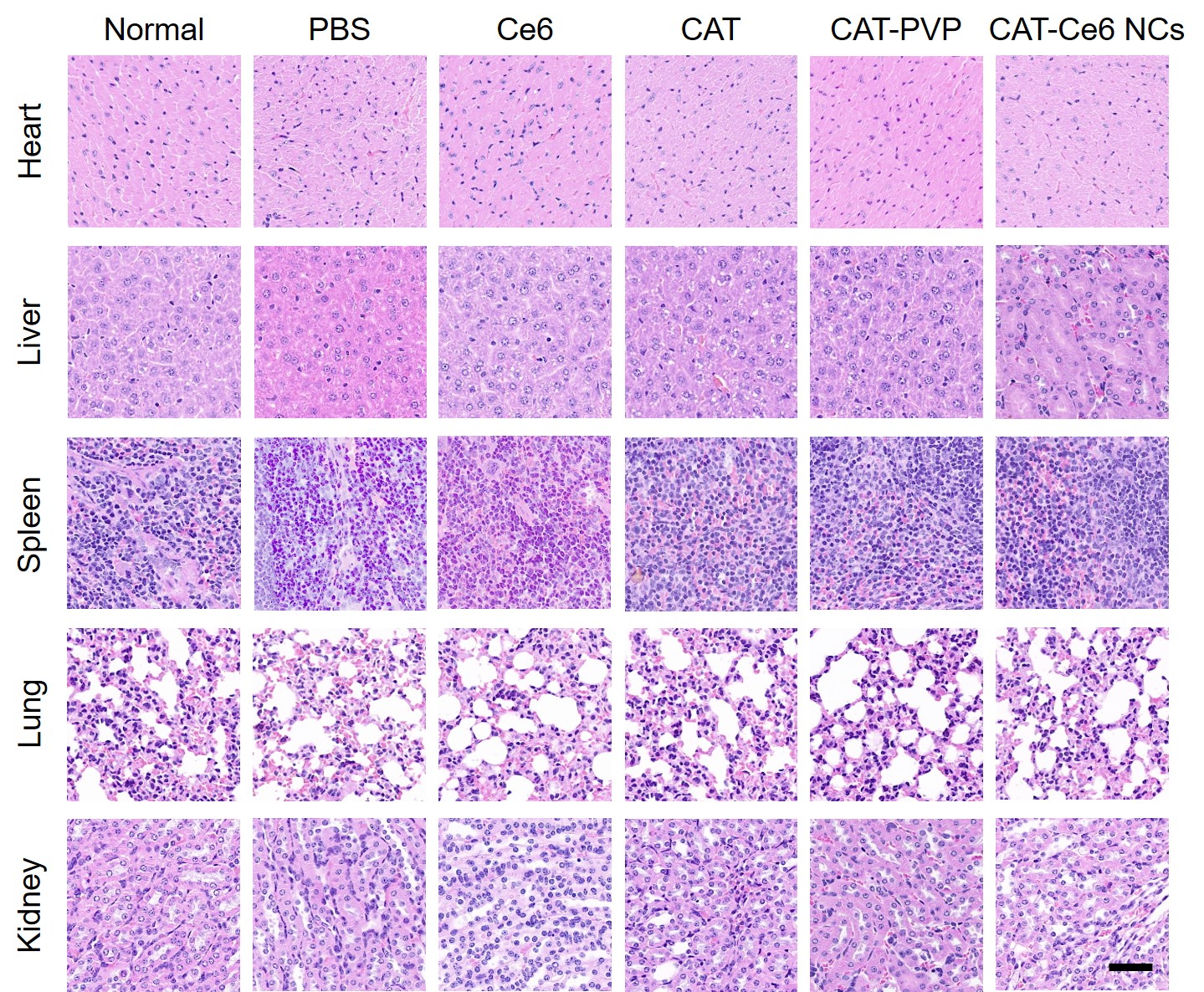


**Figure S31.** H&E staining images of main organs of mouse subcutaneously injected with PBS, CAT, Ce6, CAT-PVP, and CAT-Ce6 NCs for 12 days, respectively.


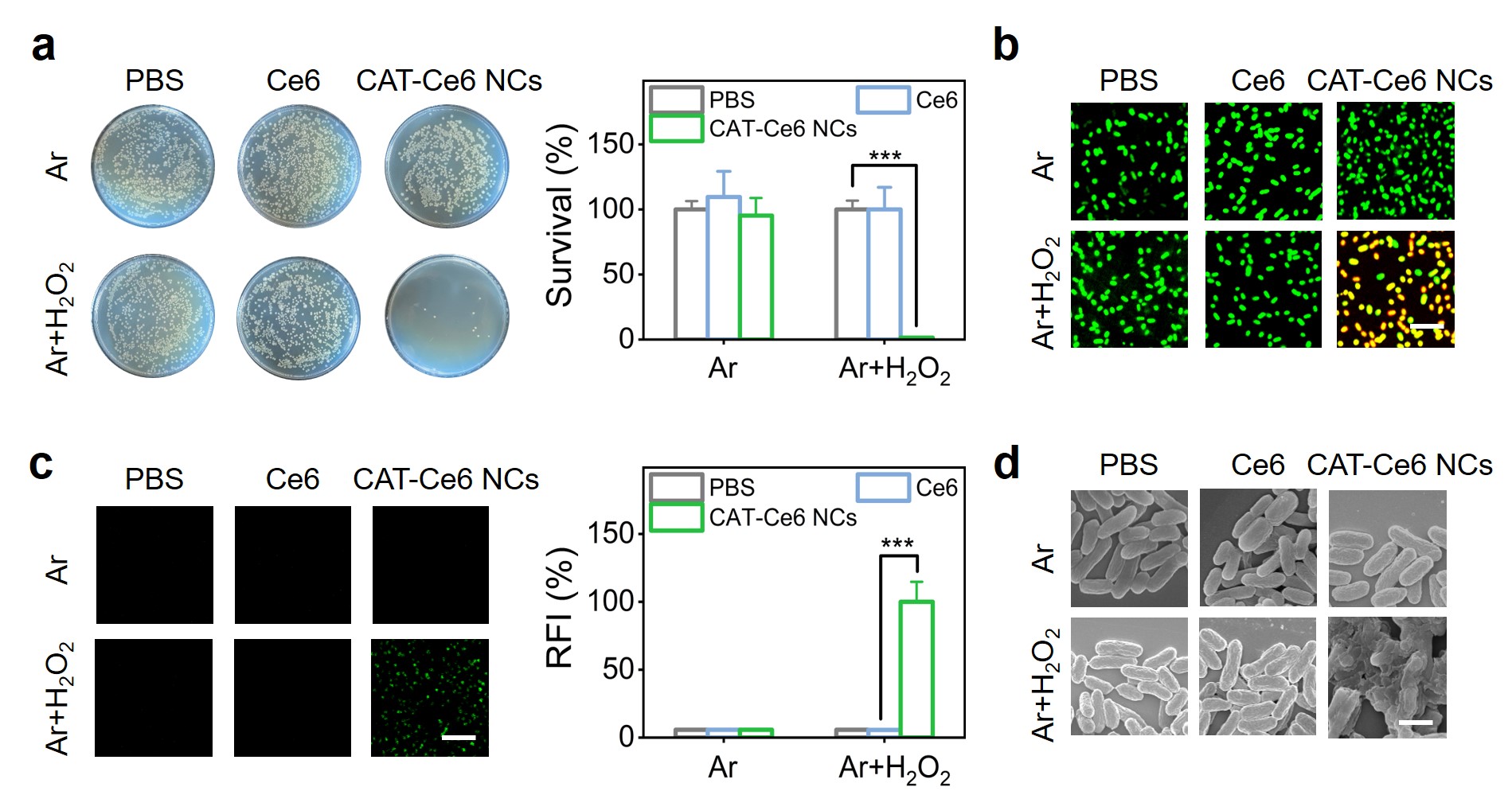


Figure S32. Photodynamic antimicrobial capability of CAT-Ce6 NCs towards *E. coli* in hypoxic condition. (a) Photographs of bacterial colonies and the corresponding quantitative survival rate in different treatment groups. (b) Confocal images of live/dead bacterial staining assay in different treatment groups in which live and dead bacteria emitted green and red fluorescence, respectively. Scale bar: 10 μm. (c) Fluorescence (green) images of intracellular ROS level and the corresponding semiquantitative statistics of relative fluorescence intensity (RFI) statistics in different treatment groups. Scale bar: 10 μm. (d) SEM-based morphology observation of bacteria in different treatment groups. Scale bar: 1 μm. Data are presented as the mean ± SD (n=3). ^***^P < 0.001.


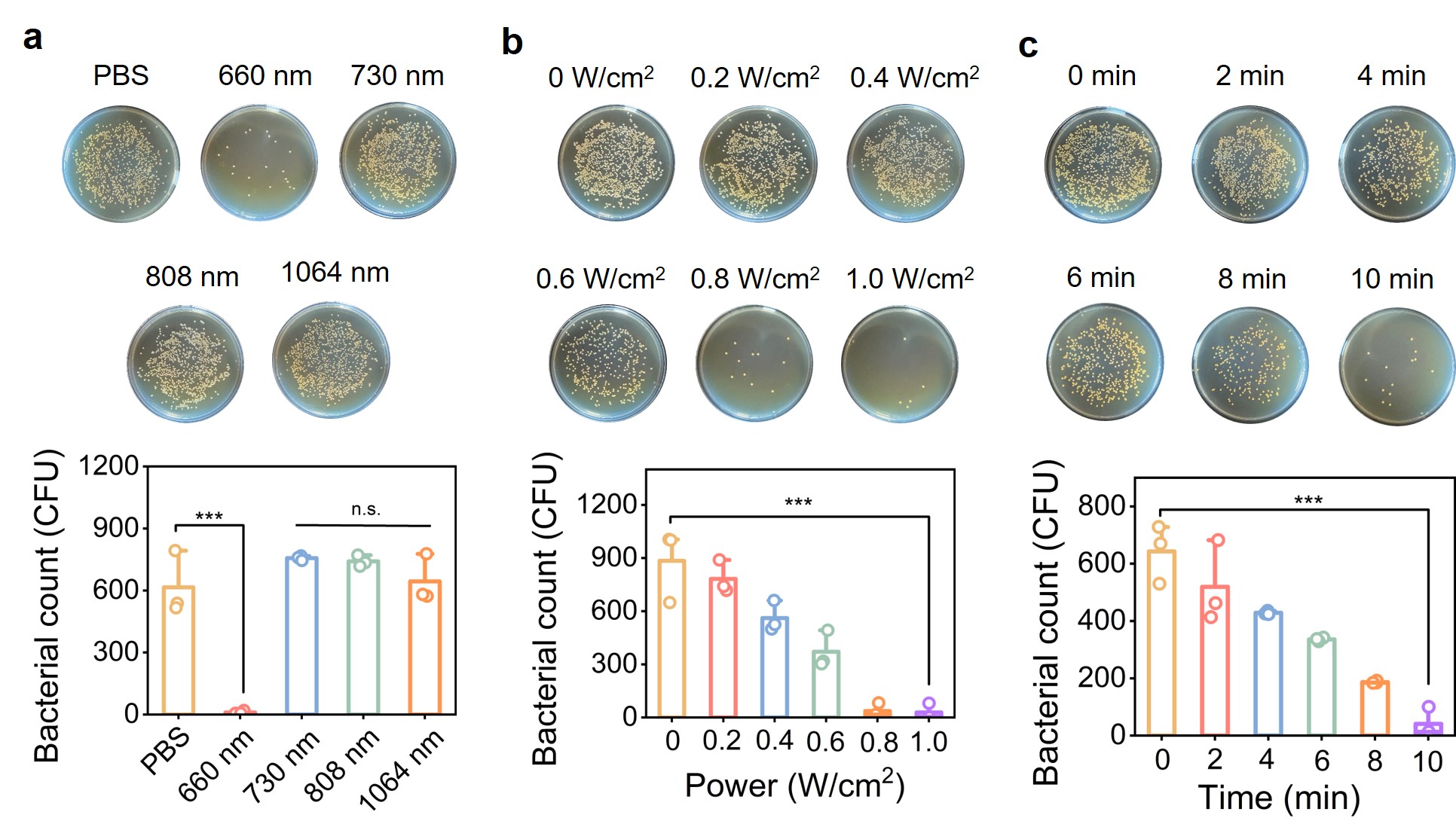


Figure S33. The antimicrobial efficiencies of CAT-Ce6 NCs toward MRSA under different PDT parameters of laser wavelength (a), power (b), and exposure time (c). Data are represented as mean ± SD (n = 3).


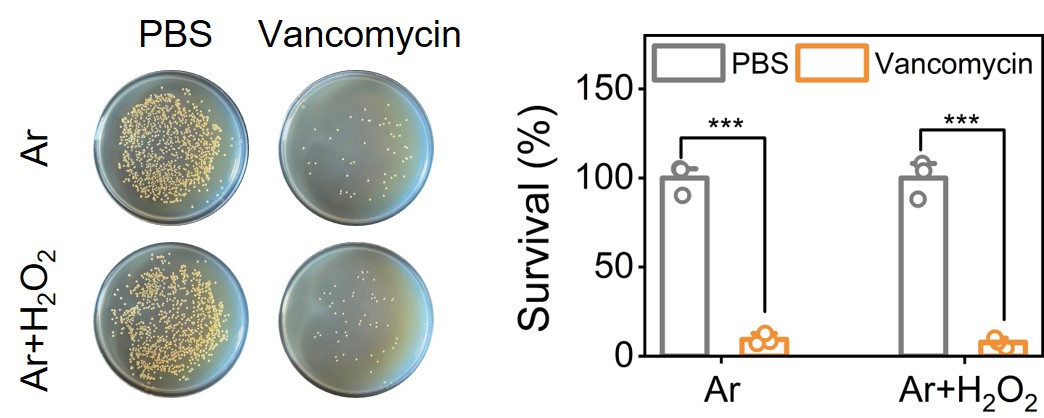


Figure S34. Photographs of bacterial colonies and the corresponding quantitative MRSA survival rate under vancomycin (2 μg/mL) treatment. Data are represented as mean ± SD (n = 3).


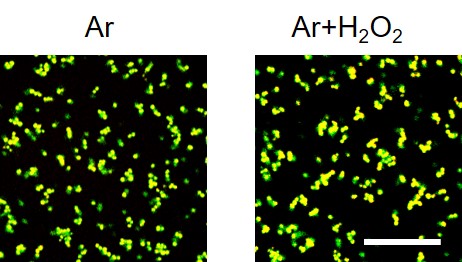


Figure S35. Live/dead bacterial staining images of MRSA under vancomycin (2 μg/mL) treatment, in which live and dead bacteria emitted green and red fluorescence, respectively. Scale bar: 10 μm.

3. Supporting Tables

Table S1. The composition of residues in each binding site of GOx-Ce6


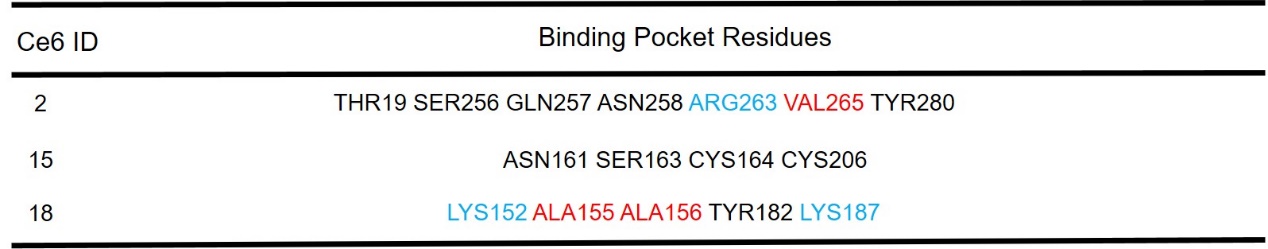


Table S2. The composition of residues in each binding site of Lys-Ce6


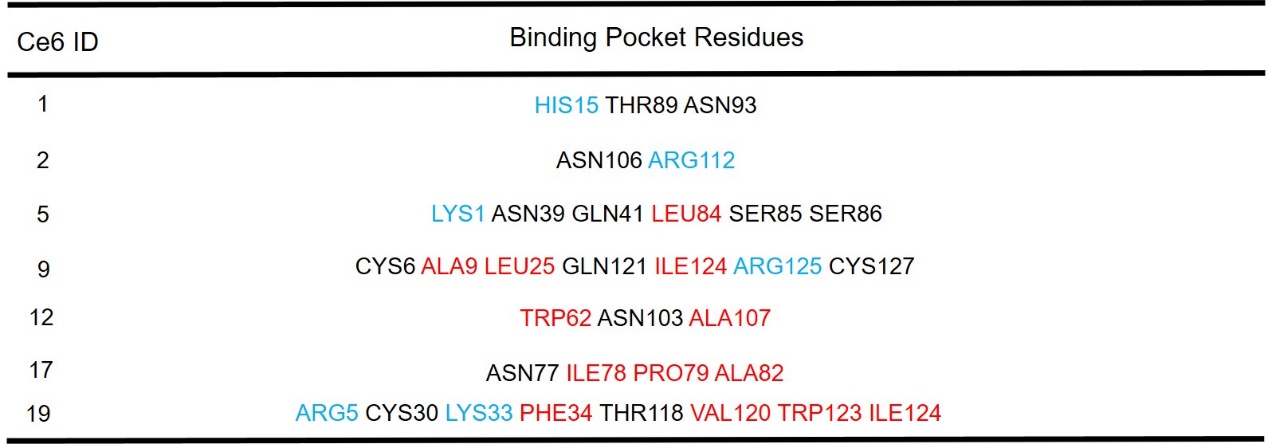


Table S3. The composition of residues in each binding site of HRP-Ce6


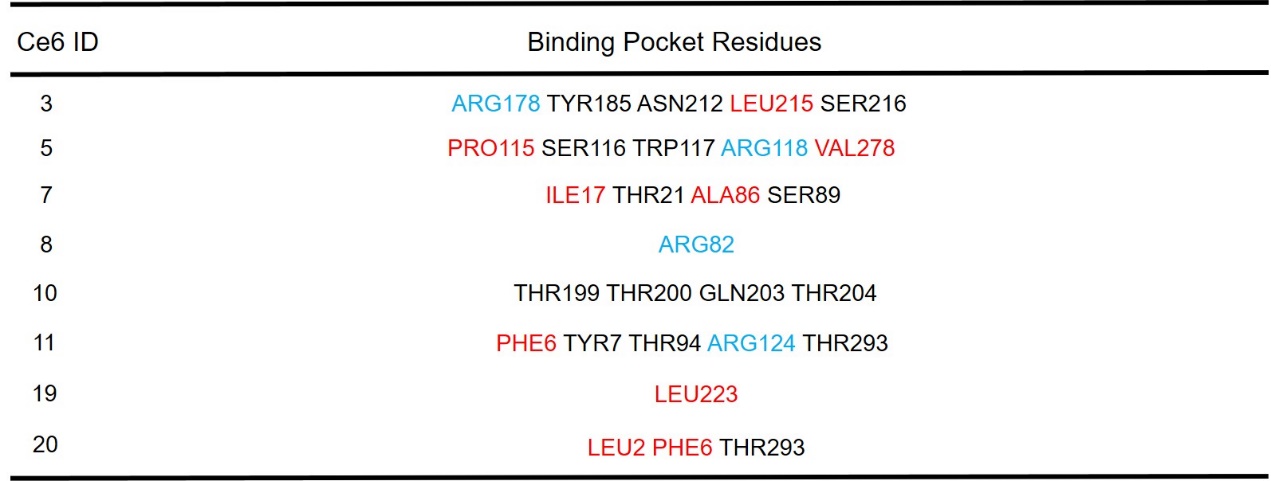


Table S4. The composition of residues in each binding site of ALP-Ce6


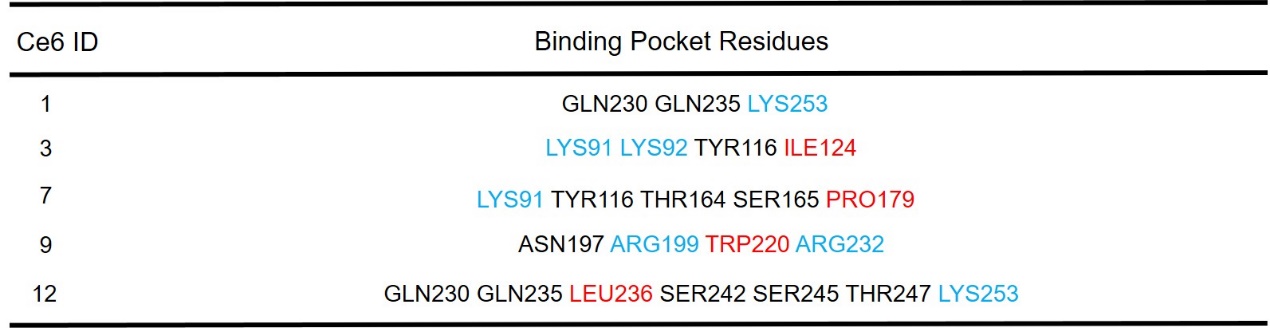


Table S5. The composition of residues in each binding site of LDHA-Ce6


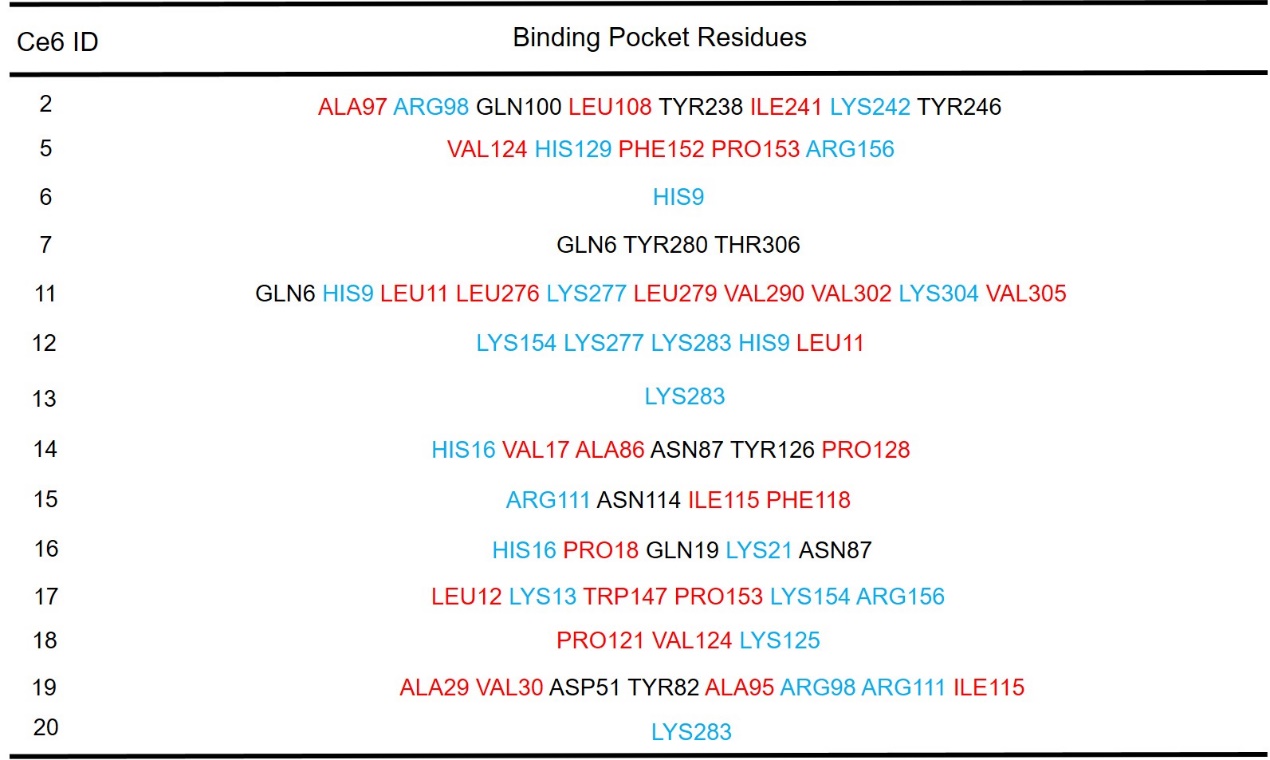


Table S6. The composition of residues in each binding site of HSA-Ce6


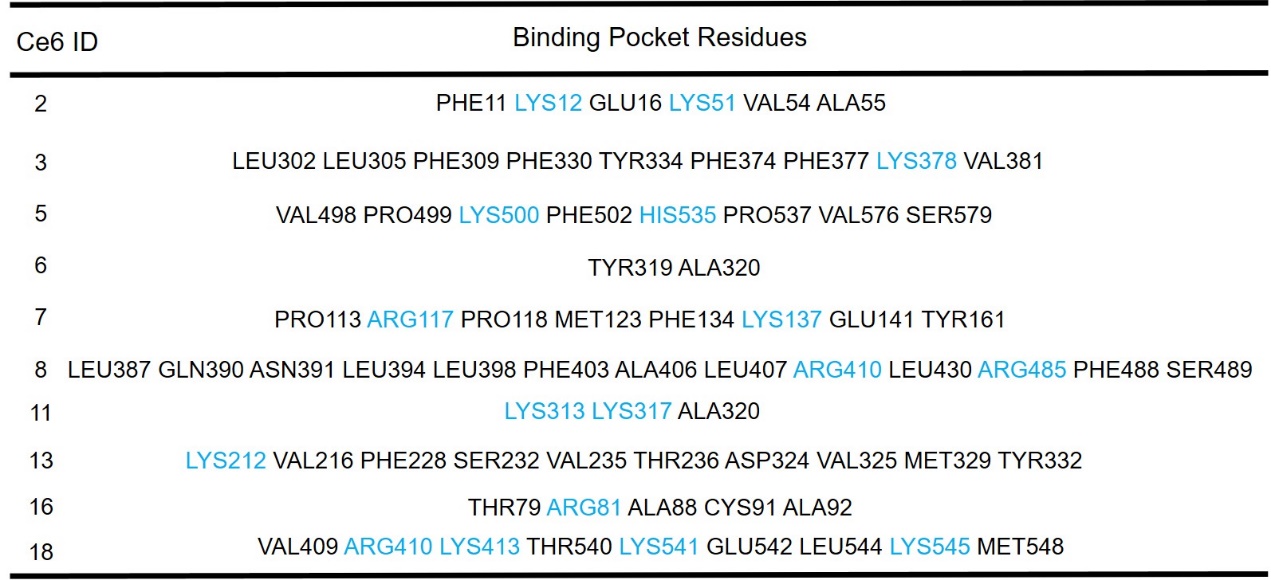

Supplement: Supplementary 1 — Experimental Section Figs. S1 to S35 Tables S1 to S6 [file research.0732.f1.docx]
